# Joint optimization of detection, task allocation, and path planning for multi-agent cooperative interception under partial observability

**DOI:** 10.1371/journal.pone.0359071

**Published:** 2026-10-13

**Authors:** Qinlong Huang, Zhikun Liu, Yasong Luo, Zhong Liu, Qingtao Xia, Miao Zhao

**Affiliations:** Naval University of Engineering, Wuhan, Hubei, P. R. China; Beijing Institute of Technology, CHINA

## Abstract

Safety-critical autonomous systems often require multiple agents to detect, assign, and intercept non-cooperative mobile targets using only partial observations. These subtasks are tightly coupled because the same agents must balance spatial exploration for detection with goal-directed motion for interception, while target maneuvers remain uncertain. This study develops and validates an integrated multi-agent coordination framework for jointly optimizing distributed detection, dynamic task allocation, and path planning under partial observability. The problem is formulated as a coupled Dec-POMDP made computationally tractable through factored decentralized policies, parameter sharing, and centralized training with decentralized execution, and a Weighted Coordinated Multi-Agent Proximal Policy Optimization (W-MAPPO) framework is introduced to adaptively coordinate competing subtasks. W-MAPPO combines an observation-conditioned Policy Ratio Predictor, an LSTM-based history-aware processor for temporal modeling of target maneuvers, and a Voronoi-based collaborative detection module for spatial coverage coordination within a centralized-training decentralized-execution paradigm. Comprehensive simulation experiments across increasing target densities compare W-MAPPO with seven representative MARL baselines and two classical methods, supported by statistical testing, ablation studies, sensitivity analysis, and representative case studies. W-MAPPO achieves a 94% success rate in the 5 vs 5 scenario and maintains stable interception performance as the number of targets increases to 10 and 15. These results demonstrate the technical reliability and practical potential of integrated subtask coordination for partially observable multi-agent systems.

## Introduction

Multi-agent systems (MAS) comprise multiple agents, each equipped with sensing, decision-making, and execution capabilities. Such systems have been widely deployed in ground search and rescue [1,2], aerial environmental monitoring [3–5], and maritime surveillance [6,7]. The rapid proliferation of autonomous platforms has simultaneously introduced new safety and security challenges: unauthorized small unmanned aerial vehicles near airports and illegal intelligent surface vessels near ports exemplify non-cooperative mobile targets that require coordinated autonomous responses. With their distributed processing, adaptive learning, and collaborative problem-solving capabilities, MAS offer a viable technical path for the dynamic protection of critical infrastructure and safety-critical areas.

Cooperative interception is a representative safety-critical multi-agent coordination problem, in which autonomous agents must respond to non-cooperative mobile targets entering an operational area and prevent them from breaching a core protection zone [8,9]. Successful execution hinges on three capabilities: rapid detection, precise task allocation, and reliable interception path planning, all operating in dynamic uncertain environments. The centralized training with decentralized execution (CTDE) paradigm [10,11] provides a natural framework for this setting: global information is exploited during training to facilitate collaborative learning, while each agent relies solely on local observations for real-time distributed decision-making.

Although existing research has explored related problems such as collaborative pursuit [12] and game-theoretic pursuit-evasion strategies [13], jointly handling multi-target interception under partial observability remains largely unexplored. Unlike fully observable settings [14], autonomous agents in partially observable environments must simultaneously perform detection, task allocation, and path planning, three subtasks whose priorities inherently conflict. Compounding this difficulty, non-cooperative targets may exhibit uncertain and reactive maneuvers [15,16] that introduce strong coupling and high uncertainty, invalidating fixed-target assumptions. These factors necessitate an integrated coordination framework capable of decoupling the composite problem while adapting to real-time environmental changes. Notably, the philosophy of jointly optimizing coupled decision variables, rather than treating them in isolation, has been validated well beyond the multi-agent domain: in reliability engineering, reinforcement-learning-based condition-based maintenance for redundant systems with arbitrary structures [17], joint optimization of quality control and maintenance policies under quality-dependent failures [18], joint maintenance and two-way stock transshipment policies for balanced systems [19], and multi-level adaptive risk control with online parameter learning [20] all demonstrate the benefits of integrated optimization of interdependent objectives, further motivating the coupled formulation adopted in this study.

Specifically, two fundamental challenges must be addressed:

1. **Detection–interception resource competition.** When the same platform serves both as sensor and interceptor, detection and interception share limited maneuvering resources and time windows. Excessive exploration delays interception opportunities; conversely, over-focusing on interception creates situational blind spots.
2. **Adversarial uncertainty.** Non-cooperative mobile targets may adopt evasive strategies whose behaviors are interdependent on the states and actions of autonomous agents, introducing strong coupling that invalidates fixed-target assumptions.

To address these challenges, we develop and validate the Weighted Coordinated Multi-Agent Proximal Policy Optimization (W-MAPPO) framework under the CTDE paradigm. W-MAPPO decouples the interception mission into three subtasks, namely detection, task allocation, and path planning, and employs a Policy Ratio Predictor to dynamically adjust subtask weights based on local observations, coupled with an LSTM-based History-Aware Processor with attention mechanism that captures target maneuvering patterns from historical trajectories to impose temporal constraints on the predicted weights.

The main contributions are summarized as follows:

1. We formulate the multi-agent cooperative interception under partial observability as a joint coordination problem (𝒫1) that explicitly couples three interdependent subtasks: distributed detection, dynamic task allocation, and interception path planning. The formulation captures the detection-interception resource competition through shared kinematic constraints and two-timescale decision variables, and characterizes uncertainty via a hybrid target maneuvering model. This coupled problem structure, which prior works circumvent by assuming known target positions or treating subtasks in isolation, is then reformulated as a Dec-POMDP amenable to multi-agent reinforcement learning.
2. We introduce W-MAPPO as an integrated subtask coordination framework rather than a single-module modification of MAPPO. The framework leverages a Policy Ratio Predictor to learn dynamic subtask weights and an LSTM-based History-Aware Processor with attention mechanism to impose temporal constraints and dynamic corrections, thereby alleviating resource competition among composite subtasks. It further incorporates a Voronoi-based collaborative detection module that coordinates spatial coverage among agents to improve detection efficiency under partial observability.
3. We evaluate the proposed framework through comprehensive and reproducible simulation experiments across multiple target-density scenarios. The evaluation compares W-MAPPO with seven representative MARL baselines and two classical coordination methods, supported by statistical significance testing, ablation studies, hyperparameter sensitivity analysis, and representative case studies. The results demonstrate that the integrated coordination framework consistently improves task success rate, interception efficiency, and spatial defense quality under partial observability.

## Related work

Existing studies related to multi-agent cooperative interception can be grouped into three streams: distributed detection under partial observability, task allocation and path planning for interception, and learning-based cooperative decision-making [21,22].

For autonomous detection in partially observable environments, coverage-based detection has been widely adopted for agent deployment [23,24]. [25] leveraged coverage control for a reach-avoid game with multiple pursuers against a single evader, though the approach is limited to single-threat scenarios. [26] developed a distributed coordination method based on a mixed coverage-tracking metric, enabling simultaneous detection and interception of multiple unknown intruders; however, the threat maneuvering model remains simplistic and does not account for adaptive evasion behaviors. More recently, [27] proposed a cross-domain collaborative exploration and coverage (CDCEC) framework for heterogeneous USV–UAV systems, integrating multi-scale grid coverage planning with a decision-tree-based adversarial strategy to achieve adaptive switching between patrol and interception modes. While comprehensive in formulation, the decision-tree-based strategy selection faces combinatorial explosion as agent count grows. Overall, existing detection-oriented studies still provide limited mechanisms for modeling how coverage behavior competes with interception behavior for the same agents’ motion resources.

Beyond detection, task allocation and path planning are central to multi-agent interception once targets are observed. [28] designed a USV-based vessel intrusion interception system for offshore wind farms that obtains target positions via real-time radar monitoring, assesses intrusion risk using the analytic hierarchy process (AHP) and entropy weight method (EWM), and plans interception paths through an improved fast marching square (FMS) algorithm. [29] introduced a priority-based dynamic resource allocation and multi-task assignment approach (P-DREAM) for perimeter defense against highly maneuvering intruders. [30] combined game theory with task allocation to enable multiple defending UAVs to cooperatively intercept attacking UAVs for area protection. These methods improve allocation or pursuit efficiency after targets are known, but they largely assume that target positions are globally available or that intruders are detected upon entering the monitored area, thereby bypassing the autonomous detection stage required under partial observability.

Reinforcement learning (RL) offers a promising avenue for online optimization in large-scale, multi-threat interception scenarios [31–33]. [34] employed MADDPG with reconstructed continuous state and action spaces and distance-based reward functions to solve multi-agent defense and attack problems. [35] proposed a modified evolutionary reinforcement learning method (MRPM) that combines differential evolution with MADDPG for cooperative region protection, improving interception success rates when defenders outnumber intruders. [36] introduced the dynamic agent-scaling framework with game-augmented reinforcement learning (DASF-GRL), which dynamically adjusts the number of defenders based on real-time threat levels and incorporates a safety barrier function from differential game theory to constrain action spaces. Despite these advances, none of these methods specifically addresses the coupling among detection, allocation, and path planning under partial observability, as they treat interception as a single-objective task without explicitly coordinating competing subtask priorities.

A final stream of related work concerns how competing objectives are weighted in multi-task learning systems. Classical approaches fix the weight of each loss term by hand, while a broad family of training-level schemes—gradient-based adaptation, homoscedastic uncertainty weighting, and gradient surgery—is reviewed in [37]. In multi-objective reinforcement learning, conflicting gradients similarly degrade policies trained under fixed preferences, and recent work develops conflict-avoidant dynamic weighting with finite-time convergence guarantees [38]. All of these operate at the training level, treating the weight vector as an optimization coefficient. The Policy Ratio Predictor in W-MAPPO differs fundamentally: the weights are part of the policy output, generated at execution time from local observations, so each agent continuously reallocates its own attention among detection, allocation, and interception as the situation evolves, and the weights are regularized toward historically consistent values through the KL-based history loss (Eq. (51)). Likewise, the role of the LSTM module is not memory augmentation of the policy, as in recurrent and sequence-model extensions of policy-gradient and value-based methods [39]; it instead serves as a consistency regularizer that infers temporally coherent weight and value estimates from the interaction history. The Voronoi module further departs from classical centralized coverage controllers in that the cell area is approximated from local samples within the communication range under partial observability and is employed as a reward signal rather than as a control law. The novelty of W-MAPPO therefore lies not in the individual modules but in their integration: converting static loss-level balancing into an execution-time, observation-conditioned, history-regularized subtask coordination mechanism that operates in concert with spatial coverage incentives.

Building on these observations, this work investigates the multi-stage cooperative interception problem under partial observability, jointly optimizing distributed detection, dynamic task allocation, and path planning to overcome the limitations of existing approaches in multi-stage task coordination and large-scale algorithmic performance.

## System modeling and problem formulation

This section establishes the system framework for multi-agent cooperative interception in adversarial environments, formalizes the three subtasks of distributed detection, dynamic task allocation, and interception path planning, and formulates the joint optimization problem. Table 1 summarizes the key notations used throughout.

**Table 1:** Main symbols used in this paper.

Symbol	Description
*M*	Number of agents
*N*	Number of threat targets
$L_{x}$	Horizontal range of task area
$L_{y}$	Vertical range of task area
*R*_core_	Radius of core area
*R*_comm_	Communication radius
*R*_detect_	Detection radius
*R*_int_	Interception radius
$v_{u}^{\max}$	Maximum speed of agent
$v_{u}^{\min}$	Minimum speed of agent
$a_{u}^{\max}$	Maximum linear acceleration of agent
$\omega_{u}^{\max}$	Maximum angular velocity of agent
$a_{\max}$	Maximum lateral acceleration of agent

### System overview

Consider the multi-agent cooperative interception system illustrated in Fig 1. The system comprises: a set of agents $U=\{u^{1},u^{2},u^{3},...,u^{M}\}$ (blue boats), each capable of detection, communication, and maneuvering interception; a set of threat targets $W=\{w^{1},w^{2},w^{3},...,w^{N}\}$ (red boats), whose maneuvering strategies are unknown and exhibit evasive behavior; a core protection area $Z_{\text{core}}=\left\{P\in {\mathbb{R}}^{2}|\left\|P\right\|\leq R_{\text{core}}\right\}$, a circular region (yellow) centered at the origin with radius *R*_core_; and a rectangular task area $Z_{\text{task}}=\left\{P=(x,y)\in {\mathbb{R}}^{2}|\left|x\right|\leq \frac{L_{x}}{2},\left|y\right|\leq \frac{L_{y}}{2}\right\}$, where $L_{x}$ and $L_{y}$ denote the horizontal and vertical extents, respectively. The center of the task area coincides with the center of the core area.

**Fig 1:**
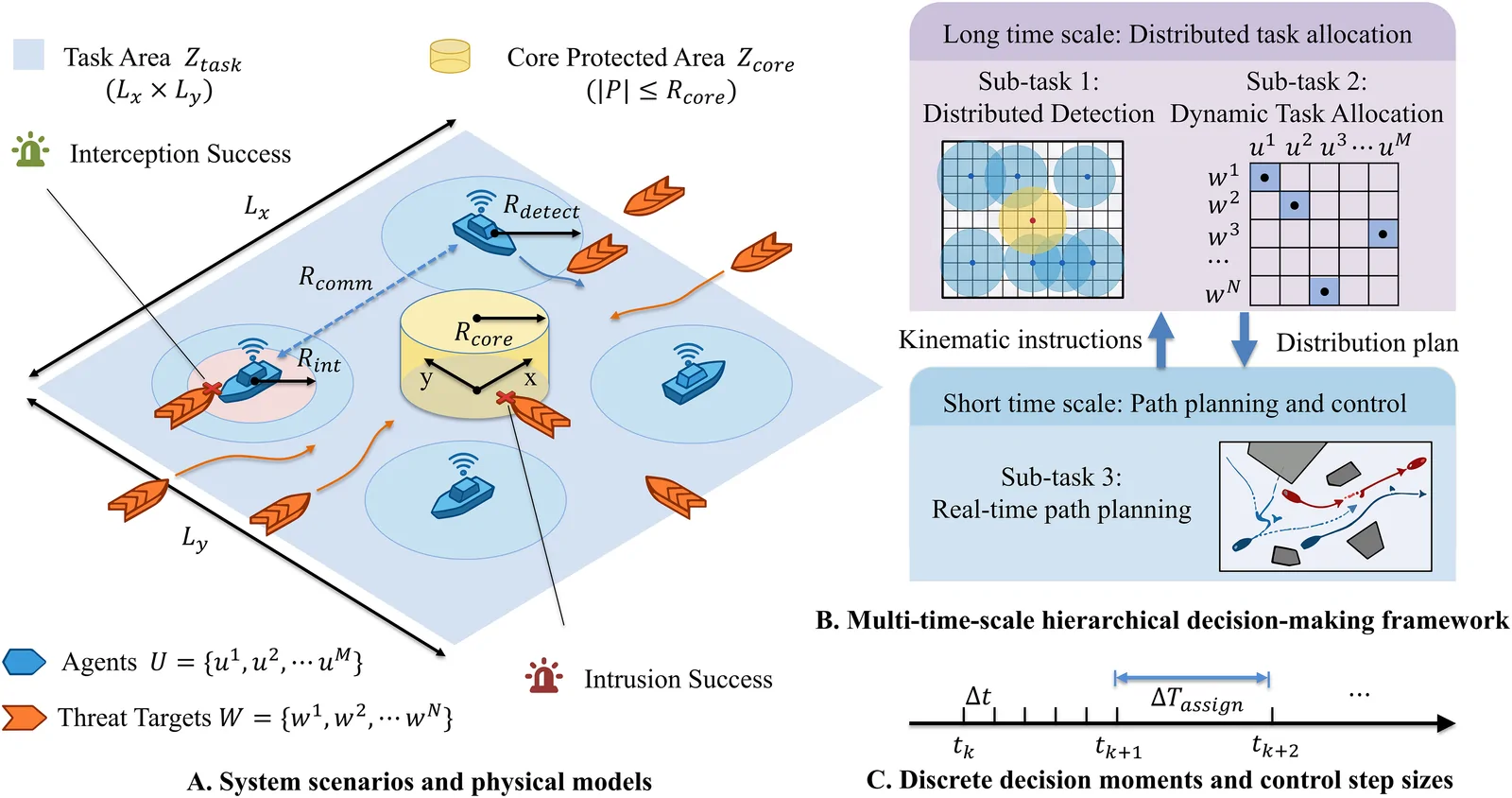
System overview of the multi-agent cooperative interception framework.

Threat targets appear randomly from outside the task area and attempt to approach its center. If any target enters *Z*_core_, the intrusion succeeds and the overall mission fails. The MAS performs a rolling interception task: upon completing one interception, an agent re-enters the next allocation round. Under limited detection and communication, the system uses distributed task allocation and path planning control. To balance responsiveness and cooperative performance, a two-timescale optimization strategy is adopted: task allocation is updated every $\Delta T_{\text{assign}}$, while motion commands are updated every control period Δt. The time axis is divided into discrete decision instants $\left\{t_{k}\right\}$ with interval $\Delta T_{\text{assign}}$, each containing multiple control steps of length Δt.

### Distributed detection

In a partially observable environment, agents cannot access global target information and must achieve situational awareness through local sensing and cooperative information sharing. The detection capability of each agent $u^{i}$ is bounded by the effective sensing radius *R*_detect_. The set of detectable targets for agent $u^{i}$ at time *t* is:


$$\begin{array}{c} {𝒟}^{i}(t)=\left\{w^{j}\in W\;|\;\left\|P_{u}^{i}(t)-P_{w}^{j}(t)\right\|\leq R_{\text{detect}}\right\}. \end{array}$$ (1)

Each agent directly obtains real-time state information (position, velocity) of targets in ${𝒟}^{i}(t)$; for targets outside this set, information is available only through communication with neighboring agents. To ensure effective coverage of the task area, agents must be spatially distributed to minimize detection blind spots. This requirement is not implemented as an independent decision variable; rather, the actual deployment emerges from the joint effect of task allocation and path planning, realized through a Voronoi-based region coverage reward in the proposed algorithm.

### Dynamic task allocation

At each decision instant $t_{k}$, the system determines a task allocation scheme based on prior information from the distributed detection module. A binary assignment variable $c_{ij}(t_{k})\in \{0,1\}$ indicates whether threat target $w^{j}$ is assigned to agent $u^{i}$ during $\left[t_{k},t_{k+1}\right)$. The assignment must satisfy:

**Single-agent load constraint**: Each agent can be assigned at most one target at the same time.


$$\begin{array}{c} \sum_{j=1}^{N}c_{ij}(t_{k})\leq 1,\;\forall i\in \{1,...,M\},\;\forall t_{k}. \end{array}$$ (2)

**Single-target assignment constraint:** Each target can be assigned to at most one agent at the same time.


$$\begin{array}{c} \sum_{i=1}^{M}c_{ij}(t_{k})\leq 1,\;\forall j\in \{1,...,N\},\;\forall t_{k}. \end{array}$$ (3)

**Distributed solution interpretation:** The assignment variables $c_{ij}(t_{k})$ describe a globally optimal allocation. In practice, however, individual agents cannot access global information. The following sections design a distributed task allocation algorithm that enables agents to cooperatively approximate this global optimum using only local detection information and limited communication (constrained by *R*_comm_).

### Path planning

At each control step Δt, agent $u^{i}$ generates continuous motion control commands based on its assigned target and local observations. The kinematic model is as follows.

Let the control variable of agent $u^{i}$ at time *t* be ${\vec{v}}_{u}^{i}(t)=[v_{u}^{i}(t),\theta_{u}^{i}(t){]}^{T}\in {\mathbb{R}}^{2}$, where $v_{u}^{i}(t)$ and $\theta_{u}^{i}(t)$ denote its speed and heading angle, respectively. The upda*t*e equation for its position $P_{u}^{i}(t)=[x_{u}^{i}(t),y_{u}^{i}(t)]$ is given by:


$$\begin{array}{c} P_{u}^{i}(t+\Delta t)=P_{u}^{i}(t)+v_{u}^{i}(t)\cdot [\cos\theta_{u}^{i}(t),\sin\theta_{u}^{i}(t){]}^{T}\cdot \Delta t. \end{array}$$ (4)

The maneuvering capability is subject to the following constraints:

**Speed limits**: $v_{u}^{\min}\leq v_{u}^{i}(t)\leq v_{u}^{\max}$.

**Angular velocity limit:** $|{\dot{\theta }}_{u}^{i}(t)|\leq \omega_{u}^{\max}$.

**Centripetal acceleration constraint:** $|{\dot{\theta }}_{u}^{i}(t)|\cdot v_{u}^{i}(t)\leq a_{\max}$, where $a_{\max}$ is the maximum lateral acceleration.

When the distance between agent $u^{i}$ and threat target $w^{j}$ falls below the interception radius *R*_int_, the target is considered successfully intercepted and removed from the scenario. The interception time $t_{\text{int}}^{j}$ and the corresponding interception distance (distance from the core area center at that moment) are recorded to evaluate defense depth. Threat targets adopt a hybrid maneuvering model encompassing directed intrusion, random walk, pulse acceleration, and feedback evasion. The detailed dynamics are provided in the Threat Target Maneuvering Model paragraph of the experimental setup section.

### Problem formulation

To evaluate the defense effectiveness of the MAS, we adopt a comprehensive defense performance metric incorporating the number of successfully intercepted targets, the number of breached targets, and the defense depth:


$$\begin{array}{c} J=N_{\text{inter}}-\alpha \cdot N_{\text{breach}}+\beta \cdot D_{\text{inter}}, \end{array}$$ (5)

where *N*_inter_ is the number of threat targets successfully intercepted by the agents during the mission, *N*_breach_ is the number of threat targets that penetrate the core protection area *Z*_core_, and *D*_inter_ is the average distance from the core area center at which the threats are intercepted (quantifying the defense depth). The coefficients α>0 and β>0 are weights that adjust the importance of different mission objectives.

Define the interception indicator $I_{\text{inter}}^{j}$ and the breach indicator $I_{\text{breach}}^{j}$ for threat target $w^{j}$ as follows:


$$\begin{array}{c} I_{\text{inter}}^{j}=\left\{ \begin{array}{cc} 1, & \exists i,t:\|P_{u}^{i}(t)-P_{w}^{j}(t)\|\leq R_{\text{int}},\\ 0, & \text{otherwise}. \end{array}\right. \end{array}$$ (6)

$$\begin{array}{c} I_{\text{breach}}^{j}=\left\{ \begin{array}{cc} 1, & \exists t:\|P_{w}^{j}(t)\|\leq R_{\text{core}}\text{ and }I_{\text{inter}}^{j}=0,\\ 0, & \text{otherwise}. \end{array}\right. \end{array}$$ (7)

Then the number of intercepted targets is $N_{\text{inter}}=\sum_{j=1}^{N}I_{\text{inter}}^{j}$, and the number of breached targets is $N_{\text{breach}}=\sum_{j=1}^{N}I_{\text{breach}}^{j}$.

For a successfully intercepted target, the interception distance is defined as:


$$\begin{array}{c} d_{\text{inter}}=\min_{t:\|P_{u}^{i}(t)-P_{w}^{j}(t)\|\leq R_{\text{int}}}\|P_{w}^{j}(t)\|. \end{array}$$ (8)

The defense depth is then given by:


$$\begin{array}{c} D_{\text{inter}}=\left\{ \begin{array}{cc} \frac{1}{N_{\text{inter}}}\sum_{j=1}^{N}d_{\text{inter}}, & N_{\text{inter}}>0,\\ 0, & N_{\text{inter}}=0. \end{array}\right. \end{array}$$ (9)

This metric directly addresses three core dimensions of the interception mission: interception capability (*N*_inter_), protection capability (*N*_breach_), and strategic quality (*D*_inter_). A sufficiently large α ensures that the cost of a single penetration far exceeds the benefit of an interception, while a reasonable β encourages interceptions to occur as far from the core area as possible.

To maximize *J* while ensuring successful subtask execution, we construct a two-timescale online optimization problem by jointly optimizing the task assignment commands $c_{ij}(t_{k})$ and motion control commands ${\vec{v}}_{u}^{i}(t)$:


$$\begin{array}{cc} 𝒫1:\;\max_{\left\{c_{ij}(t_{k}),{\vec{v}}_{u}^{i}(t)\right\}}\; & \lim_{t\to \infty }J\\ \text{s.t.}\; & |x_{u}^{i}(t)|\leq \frac{L_{x}}{2},\;|y_{u}^{i}(t)|\leq \frac{L_{y}}{2}\;\text{(a)}\\ & {𝒟}^{i}(t)=\left\{w^{j}\in W\;|\;\|P_{u}^{i}(t)-P_{w}^{j}(t)\|\leq R_{\text{detect}}\right\}\;\text{(b)}\\ & {𝒩}^{i}(t)=\left\{u^{k}\in U\;|\;\|P_{u}^{i}(t)-P_{u}^{k}(t)\|\leq R_{\text{comm}},\;i\;=\;\;\;\;/\;k\right\}\;\text{(c)}\\ & \sum_{j=1}^{N}c_{ij}(t_{k})\leq 1,\;\forall i\in \{1,...,M\},\;\forall t_{k}\;\text{(d)}\\ & \sum_{i=1}^{M}c_{ij}(t_{k})\leq 1,\;\forall j\in \{1,...,N\},\;\forall t_{k}\;\text{(e)}\\ & v_{u}^{\min}\leq v_{u}^{i}(t)\leq v_{u}^{\max},\;|{\dot{\theta }}_{u}^{i}(t)|\leq \omega_{u}^{\max},\;|{\dot{\theta }}_{u}^{i}(t)|\cdot v_{u}^{i}(t)\leq a_{\max}\;\text{(f)} \end{array}$$ (10)

Constraint (10a) confines agents within the task area. Constraints (10b) and (10c) formalize the limited detection and communication ranges, respectively. Constraints (10d)–(10f) bound the task assignment and motion control variables as defined above.

Since 𝒫1 exhibits partial observability, multi-stage decision making, and a hybrid continuous-discrete action space, traditional analytical methods cannot solve it directly. We therefore reformulate 𝒫1 as a Dec-POMDP to enable policy optimization via reinforcement learning.

We note that general Dec-POMDPs are NEXP-complete, so exact solutions of the coupled formulation are intractable. W-MAPPO adopts five standard mechanisms that jointly make the problem tractable in practice. First, under CTDE the joint policy factorizes as $\pi (a(t)|o(t))=\prod_{i=1}^{M}\pi^{i}(a^{i}(t)|o^{i}(t))$, so execution-time decision complexity grows linearly, rather than exponentially, in *M*. Second, parameter sharing across homogeneous agents compresses the joint policy space to a single policy. Third, the centralized critic is used only during training, moving the credit-assignment difficulty to the offline phase. Fourth, the two-timescale structure reduces the effective branching factor: allocation commands are selected only at decision instants $\Delta T_{\text{assign}}$ apart, while motion commands are optimized at the finer control period Δt. Fifth, policy-gradient optimization provides approximate rather than exact optimality; accordingly, the stability analysis in the W-MAPPO section characterizes an approximate stationary region rather than claiming global optimality.

## Dec-POMDP formulation

We reformulate 𝒫1 as a Dec-POMDP represented by the tuple ⟨𝒮,𝒪,𝒜,P,R⟩, where 𝒮 is the global state space, $𝒪=\{o^{i}(t){\}}_{i=1}^{M}$ is the joint observation space, $𝒜=\{a^{i}(t){\}}_{i=1}^{M}$ is the joint action space, 𝒫:𝒮×𝒜×𝒮→ℝ is the state transition model, and ℛ:𝒮×𝒜→ℝ is the reward function.

### State space

The global state $S(t)=\{S_{U}(t),S_{W}(t),S_{T}(t)\}$ aggregates the agent kinematic states $X_{u}^{i}(t)$, threat target states $X_{w}^{j}(t)$, and task-level information $S_{T}^{i}(t)=\{T_{a}^{i}(t),{𝒟}^{i}(t),{𝒩}^{i}(t),{ℐ}^{i}(t)\}$ as defined in the System Modeling and Problem Formulation section. This state is available only during centralized training; individual agents cannot observe it directly.

### Observation space

Agent $u^{i}$ cannot access the global state and relies on local observation $o^{i}(t)$ comprising four components:

- **Self-state** $o_{\text{self}}^{i}(t)=[x_{u}^{i},y_{u}^{i},v_{u}^{i},\theta_{u}^{i},T_{a}^{i}]\in {\mathbb{R}}^{5}$.
- **Self-detection** $o_{\text{detect}}^{i}(t)$: states of up to *N*’ detected targets within *R*_detect_, zero-padded. Dimension: 4*N*.
- **Neighbor status** $o_{\text{neighbor}}^{i}(t)$: states and detection information of the $K_{\max}$ nearest neighbors within *R*_comm_. Dimension: $(4N+5)K_{\max}$.
- **Voronoi zone** $o_{\text{voronoi}}^{i}(t)$: relative positions to $K_{\max}$ neighbors, encoding the approximate Voronoi responsibility boundary. Dimension: $2K_{\max}$.

The total observation dimension is $\text{Dim}(𝒪)=5+4N+(4N+5)K_{\max}+2K_{\max}$

### Action space

The action $a^{i}(t)=\{C^{i}(t),{\vec{v}}_{c}^{\;i}(t)\}$ comprises a discrete task allocation command and a continuous motion control command.

The task allocation $C^{i}(t)\in \{0,1,...,N^{*}\}$ is updated at decision instants $\left\{t_{k}\right\}$ with interval $\Delta T_{\text{assign}}$, where $C^{i}(t)=j$ (j≥1) assigns agent $u^{i}$ to intercept target $w^{j}$, and $C^{i}(t)=0$ indicates no assignment. The motion control ${\vec{v}}_{c}^{\;i}(t)=(v_{c}^{i}(t),\theta_{c}^{i}(t))\in {\mathbb{R}}^{2}$ is updated at each control step Δt, subject to:


$$\begin{array}{c} v_{c}^{\min}\leq v_{c}^{i}(t)\leq v_{c}^{\max},\;\left|{\dot{\theta }}_{c}^{i}(t)\right|\leq \omega_{c}^{\max},\;\left|{\dot{\theta }}_{c}^{i}(t)\right|\cdot v_{c}^{i}(t)\leq a_{\max}. \end{array}$$ (11)

### Reward function

Building on the interception problem model, we design a multistage hybrid reward function based on task decomposition and auxiliary guidance. The total reward for agent $u^{i}$ at time *t* is


$$\begin{array}{c} R^{i}(t)=\rho_{\text{det}}(t)\cdot R_{\text{det}}^{i}(t)+\rho_{\text{ass}}(t)\cdot R_{\text{ass}}^{i}(t)+\rho_{\text{int}}(t)\cdot R_{\text{int}}^{i}(t), \end{array}$$ (12)

where $R_{\text{det}}^{i}(t)$, $R_{\text{ass}}^{i}(t)$, and $R_{\text{int}}^{i}(t)$ are the reward functions for cooperative detection, task allocation, and interception subtasks, with $\rho_{\text{det}}(t)$, $\rho_{\text{ass}}(t)$, and $\rho_{\text{int}}(t)$ as their dynamic weights. These weights are generated by the weight adjustment module from the agent’s local observation and satisfy nonnegativity and sum-to-one constraints:


$$\begin{array}{c} \rho_{\text{det}}(t)\geq 0,\;\rho_{\text{ass}}(t)\geq 0,\;\rho_{\text{int}}(t)\geq 0,\;\rho_{\text{det}}(t)+\rho_{\text{ass}}(t)+\rho_{\text{int}}(t)=1. \end{array}$$ (13)

#### Cooperative detection reward.

The cooperative detection reward $R_{\text{det}}^{i}(t)$ for agent $u^{i}$ comprises three components:


$$\begin{array}{c} R_{\text{det}}^{i}(t)=r_{\text{det}}^{i}(t)+r_{\text{exp}}^{i}(t)+r_{\text{vor}}^{i}(t), \end{array}$$ (14)

where $r_{\text{det}}^{i}(t)$, $r_{\text{exp}}^{i}(t)$, and $r_{\text{vor}}^{i}(t)$ denote the new target discovery reward, area exploration reward, and Voronoi collaboration reward, respectively.

$r_{\text{det}}^{i}(t)$ recognizes agents for discovering new threat targets:


$$\begin{array}{c} r_{\text{det}}^{i}(t)=\alpha_{\text{det}}N_{\text{new}}^{i}(t), \end{array}$$ (15)

where $\alpha_{\text{det}}$ is a one-time reward coefficient for discovering a new target. $N_{\text{new}}^{i}(t)$ is the number of newly detected threat targets by agent $u^{i}$ at time *t*:


$$\begin{array}{c} N_{\text{new}}^{i}(t)=|{𝒟}^{i}(t)⧵{𝒟}^{i}(t-1)|. \end{array}$$ (16)

$r_{\text{exp}}^{i}(t)$ encourages exploration of rarely visited areas:


$$\begin{array}{c} r_{\text{exp}}^{i}(t)=\frac{\alpha_{\text{exp}}}{\ln(\beta_{\text{exp}}+\tau_{t}^{i}(g)+\sum_{k\in {𝒩}_{K}^{i}(t)}\tau_{t}^{k}(g))}, \end{array}$$ (17)

where $g=𝒢(P_{u}^{i}(t))$ is the grid index corresponding to the agent’s current position, $\tau_{t}^{i}(g)$ denotes the cumulative number of visits by agent $u^{i}$ to grid *g* up to time *t*, ${𝒩}_{K}^{i}(t)$ represents the set of neighbors of agent $u^{i}$ at time *t*, $\alpha_{\text{exp}}$ is the scaling coefficient for area exploration reward, and $\beta_{\text{exp}}$ is a tuning coefficien*t* for the initial exploration reward. *Z*_task_ is divided into $N_{x}\times N_{y}$ grids, each of size $\left(L_{x}/N_{x})\times (L_{y}/N_{y}\right)$. The *g*rid mapping rule is


$$\begin{array}{c} 𝒢(x,y)=(\lfloor \frac{N_{x}}{L_{x}}(x+\frac{L_{x}}{2})\rfloor ,\;\lfloor \frac{N_{y}}{L_{y}}(y+\frac{L_{y}}{2})\rfloor ), \end{array}$$ (18)

where coordinates are mapped to grids via floor rounding. The visit record is updated as


$$\begin{array}{cc} \tau_{t+1}^{i}(g) & =\tau_{t}^{i}(g)+\delta_{g}(t), \end{array}$$ (19)

$$\begin{array}{cc} \delta_{g}(t) & =\left\{ \begin{array}{cc} 1, & \text{if }g=𝒢(P_{u}^{i}(t)),\\ 0, & \text{otherwise}. \end{array}\right. \end{array}$$ (20)

This component provides the highest reward for the first visit and diminishing returns for repeated visits.

$r_{\text{vor}}^{i}(t)$ encourages each agent’s Voronoi cell area to approach the ideal distribution:


$$\begin{array}{c} r_{\text{vor}}^{i}(t)=\alpha_{\text{vor}}\cdot (1-\frac{\left|A_{\text{local}}^{i}(t)-A_{\text{ideal}}\right|}{A_{\text{ideal}}}), \end{array}$$ (21)

where $\alpha_{\text{vor}}$ is the Voronoi responsibility zone collaboration reward coefficient. The approximate area of the Voronoi cell of agent $u^{i}$ at time *t* is


$$\begin{array}{c} A_{\text{local}}^{i}(t)=\frac{\pi R_{\text{comm}}^{2}}{N_{s}}\sum_{k=1}^{N_{s}}I_{\text{vor}}^{i}(x^{k}), \end{array}$$ (22)

where $N_{s}$ points are sampled within the agent’s communication range, while $I_{\text{vor}}^{i}(x^{k})$ is an indicator function that determines whether a sampled point $x^{k}$ belongs to the Voronoi cell. The sampling count $N_{s}=50$ (Table 4) is chosen to balance approximation accuracy and computational cost; preliminary tests showed that $N_{s}\geq 30$ yields stable area estimates with <5% variance:


$$\begin{array}{c} I_{\text{vor}}^{i}(x^{k})=\left\{ \begin{array}{cc} 1, & \text{if }\|x^{k}-P_{u}^{i}(t)\|\leq \|x^{k}-P_{u}^{j}(t)\|,\;\forall j\in {𝒩}_{K}^{i}(t),\\ 0, & \text{otherwise}. \end{array}\right. \end{array}$$ (23)

As shown in Fig 2, the Voronoi cell’s area is approximated by determining the proportion of sampling points that belong to the Voronoi region of this agent.

**Fig 2:**
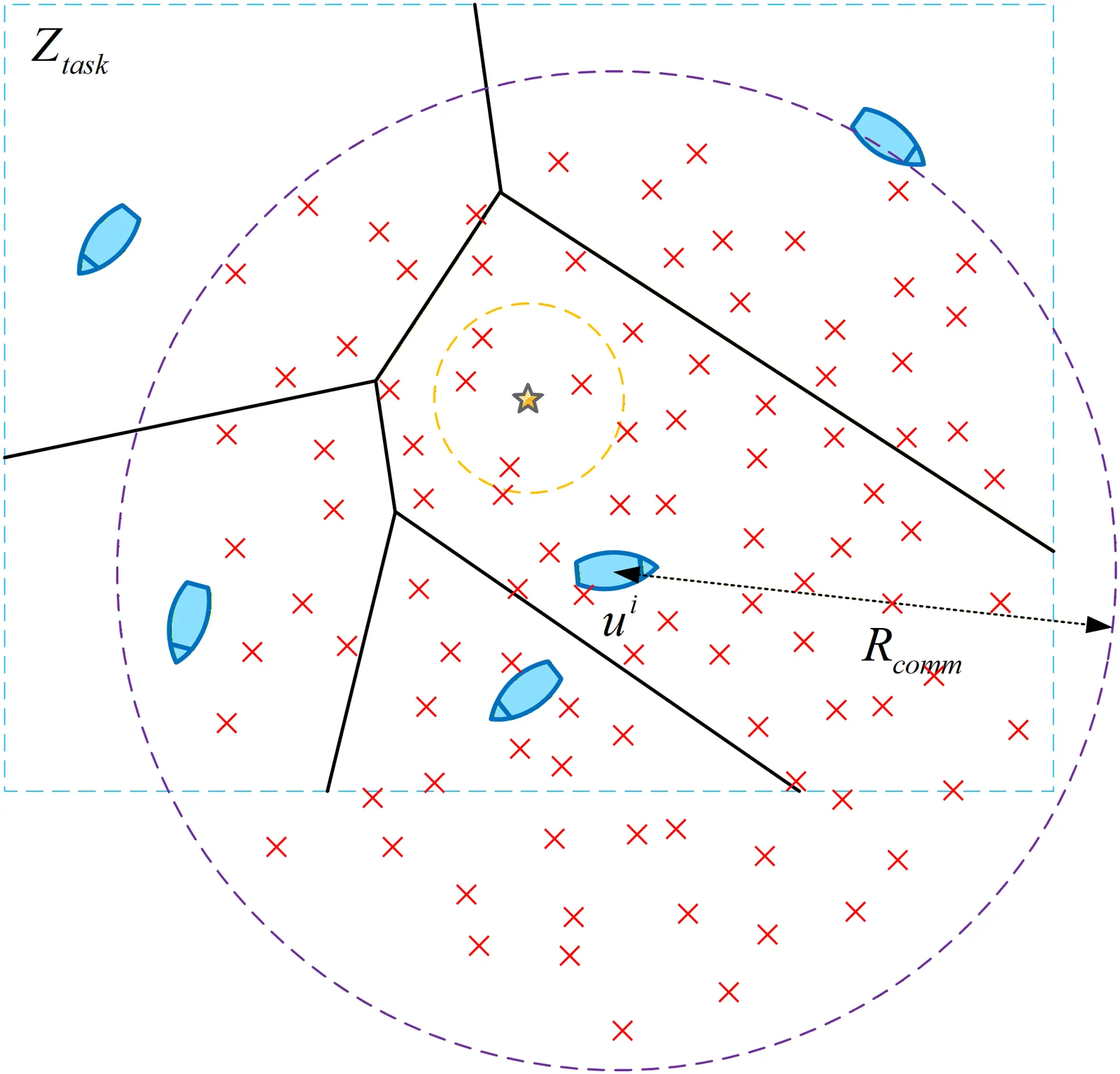
Random sampling for the approximate calculation of the Voronoi cell’s area.

The ideal Voronoi cell area under uniform distribution is


$$\begin{array}{c} A_{\text{ideal}}=\frac{L_{x}\cdot L_{y}}{M}, \end{array}$$ (24)

where *M* is the total number of agents.

#### Task allocation reward.

The task allocation reward $R_{\text{ass}}^{i}(t)$ for agent $u^{i}$ comprises three components:


$$\begin{array}{c} R_{\text{ass}}^{i}(t)=r_{\text{dis}}^{i}(t)+r_{\text{comp}}^{i}(t)+r_{\text{switch}}^{i}(t), \end{array}$$ (25)

where $r_{\text{dis}}^{i}(t)$, $r_{\text{comp}}^{i}(t)$, and $r_{\text{switch}}^{i}(t)$ are the distance matching reward, distributed competition penalty, and switching penalty, respectively.

$r_{\text{dis}}^{i}(t)$ encourages the assignment of closer targets using an exponential decay form:


$$\begin{array}{c} r_{\text{dis}}^{i}(t)=\alpha_{\text{dis}}\cdot \exp(-\frac{d_{\text{ass}}^{i}(t)}{d_{\text{ref}}})\cdot 1_{\left\{C^{i}(t)\geq 1\right\}}, \end{array}$$ (26)

where $d_{\text{ass}}^{i}(t)=\|P_{u}^{i}(t)-P_{w}^{C^{i}(t)}(t)\|$ is the distance between agent $u^{i}$ and its assigned threat target $w^{C^{i}(t)}$, *d*_ref_ is a reference distance for the matching reward, $1_{\left\{\cdot \right\}}$ is the indicator function (1 if the condition is true; 0 if otherwise), and $\alpha_{\text{dis}}$ is the scaling coefficient for distance matching.

$r_{\text{comp}}^{i}(t)$ penalizes allocation decisions that conflict with neighbors:


$$\begin{array}{c} r_{\text{comp}}^{i}(t)=-\beta_{\text{comp}}[\exp(|{𝒞}_{\text{local}}^{i}(t)|)-1], \end{array}$$ (27)

where ${𝒞}_{\text{local}}^{i}(t)=\{j\in {𝒩}_{K}^{i}(t)|C^{i}(t)=C^{j}(t)\geq 1\}$ is the local competition conflict set (representing the set of neighboring agents assigned to the same target as agent $u^{i}$), $\left|{𝒞}_{\text{local}}^{i}(t)\right|$ denotes the number of competitors observed locally, and $\beta_{\text{comp}}$ is the competition penalty scaling coefficient.

$r_{\text{switch}}^{i}(t)$ penalizes frequent task switching to maintain allocation stability:


$$\begin{array}{c} r_{\text{switch}}^{i}(t)=-\beta_{\text{switch}}1_{\left\{C^{i}(t)\neq C^{i}(t-1)\right\}}\delta_{\text{switch}}(C^{i}(t),C^{i}(t-1)), \end{array}$$ (28)

where $\beta_{\text{switch}}$ is the switching penalty scaling coefficient. $\delta_{\text{switch}}$ is the task switching severity function:


$$\begin{array}{c} \delta_{\text{switch}}(a,b)=\left\{ \begin{array}{cc} 1, & \text{if }a\geq 1,b\geq 1\text{ and }a\neq b,\\ 0.5, & \text{if }a=0\text{ and }b\geq 1,\\ 0.2, & \text{if }a\geq 1\text{ and }b=0,\\ 0, & \text{otherwise}, \end{array}\right. \end{array}$$ (29)

where $1_{\left\{\cdot \right\}}$ is the indicator function. The severity function assigns different penalty levels according to the switching pattern: a full penalty (1.0) for switching between different active tasks, a moderate penalty (0.5) for abandoning an active task, a light penalty (0.2) for resuming a task, and no penalty (0) otherwise.

#### Path planning reward.

The path planning reward $R_{\text{int}}^{i}(t)$ for agent $u^{i}$ comprises three components:


$$\begin{array}{c} R_{\text{int}}^{i}(t)=r_{\text{appr}}^{i}(t)+r_{\text{angle}}^{i}(t)+r_{\text{inter}}^{i}(t), \end{array}$$ (30)

where $r_{\text{appr}}^{i}(t)$, $r_{\text{angle}}^{i}(t)$, and $r_{\text{inter}}^{i}(t)$ are the approach reward, angular advantage reward, and successful path planning reward, respectively.

$r_{\text{appr}}^{i}(t)$ encourages the agent to move closer to its assigned target, with the reward increasing as the distance decreases:


$$\begin{array}{c} r_{\text{appr}}^{i}(t)=\alpha_{\text{appr}}\exp(-\frac{d_{\text{ass}}^{i}(t)}{R_{\text{detect}}}), \end{array}$$ (31)

where $d_{\text{ass}}^{i}(t)=\|P_{u}^{i}(t)-P_{w}^{C^{i}(t)}(t)\|$ is the distance between agent $u^{i}$ and its assigned threat target $w^{C^{i}(t)}$, *R*_detect_ is the detection radius, and $\alpha_{\text{appr}}$ is the approach reward scaling coefficient.

$r_{\text{angle}}^{i}(t)$ encourages the agent to enter the angular advantage region for effective interception:


$$\begin{array}{c} r_{\text{angle}}^{i}(t)=\alpha_{\text{angle}}\sin^{2}(\frac{\theta_{a}}{2}), \end{array}$$ (32)

where $\theta_{a}\in (0,\pi ]$ is the interception advantage angle and $\alpha_{\text{angle}}$ is the angular advantage reward scaling coefficient.

In Fig 3, the angular advantage region for intercepting threat target $w^{j}$ is denoted by a red circular area, with the line connecting $w^{j}$ and the protected zone center as its diameter. The interception advantage angle $\theta_{a}$ is formed by the threat target, the agent, and the task area center. When the agent lies inside the advantage region ($\theta_{a}$ obtuse), it approaches the target from the front or side-front, reducing the target’s evasion space. When $\theta_{a}$ is acute, the agent is outside the advantage region, leading to tail-chasing and reduced interception success.

**Fig 3:**
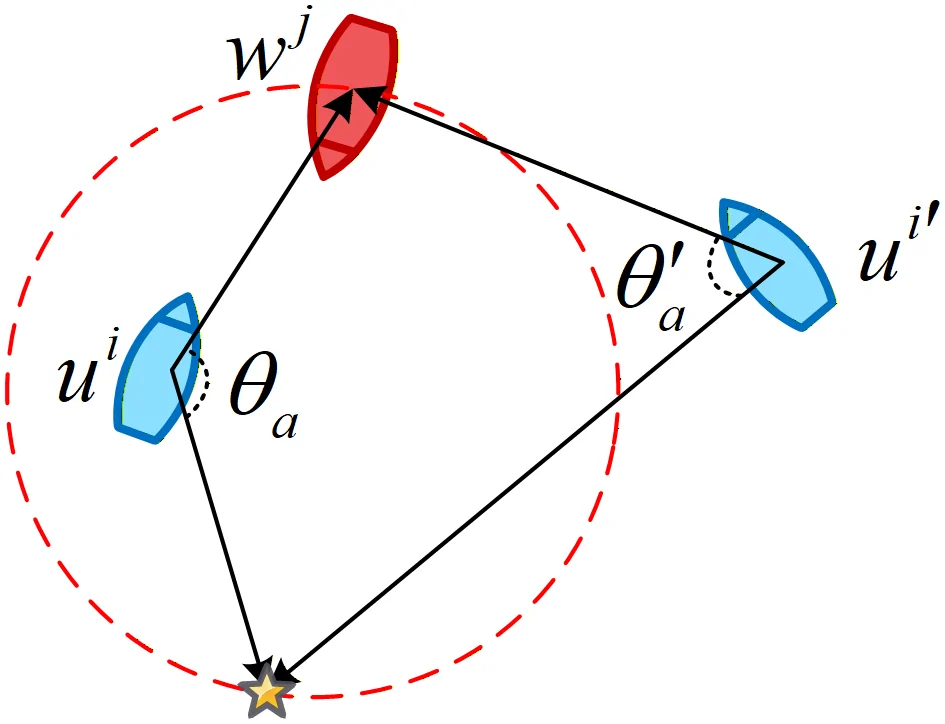
Schematic of the interception advantage angle.

$r_{\text{inter}}^{i}(t)$ provides a high reward for successful interception:


$$\begin{array}{c} r_{\text{inter}}^{i}(t)=\alpha_{\text{inter}}I_{\text{inter}}^{i}(t), \end{array}$$ (33)

where $\alpha_{\text{inter}}$ is the one-time reward coefficient for successful interception. $I_{\text{inter}}^{i}(t)$ is the interception indicator function:


$$\begin{array}{c} I_{\text{inter}}^{i}(t)=\left\{ \begin{array}{cc} 1, & \text{if }\exists \;w^{j}\in W:\|P_{u}^{i}(t)-P_{w}^{j}(t)\|\leq R_{\text{int}},\\ 0, & \text{otherwise}, \end{array}\right. \end{array}$$ (34)

where *R*_int_ is the interception radius. This component provides a one-time high reward when agent $u^{i}$ successfully intercepts any threat target, encouraging the agent to accomplish the final interception task.

## W-MAPPO algorithm design

This section presents the W-MAPPO algorithm, including its system architecture, network structure, and training procedure. Built upon the CTDE paradigm, the algorithm facilitates efficient collaborative training while preserving fully decentralized execution.

### System architecture

W-MAPPO’s architecture comprises two modules: the decentralized execution module and the centralized training module (Fig 4).

**Fig 4:**
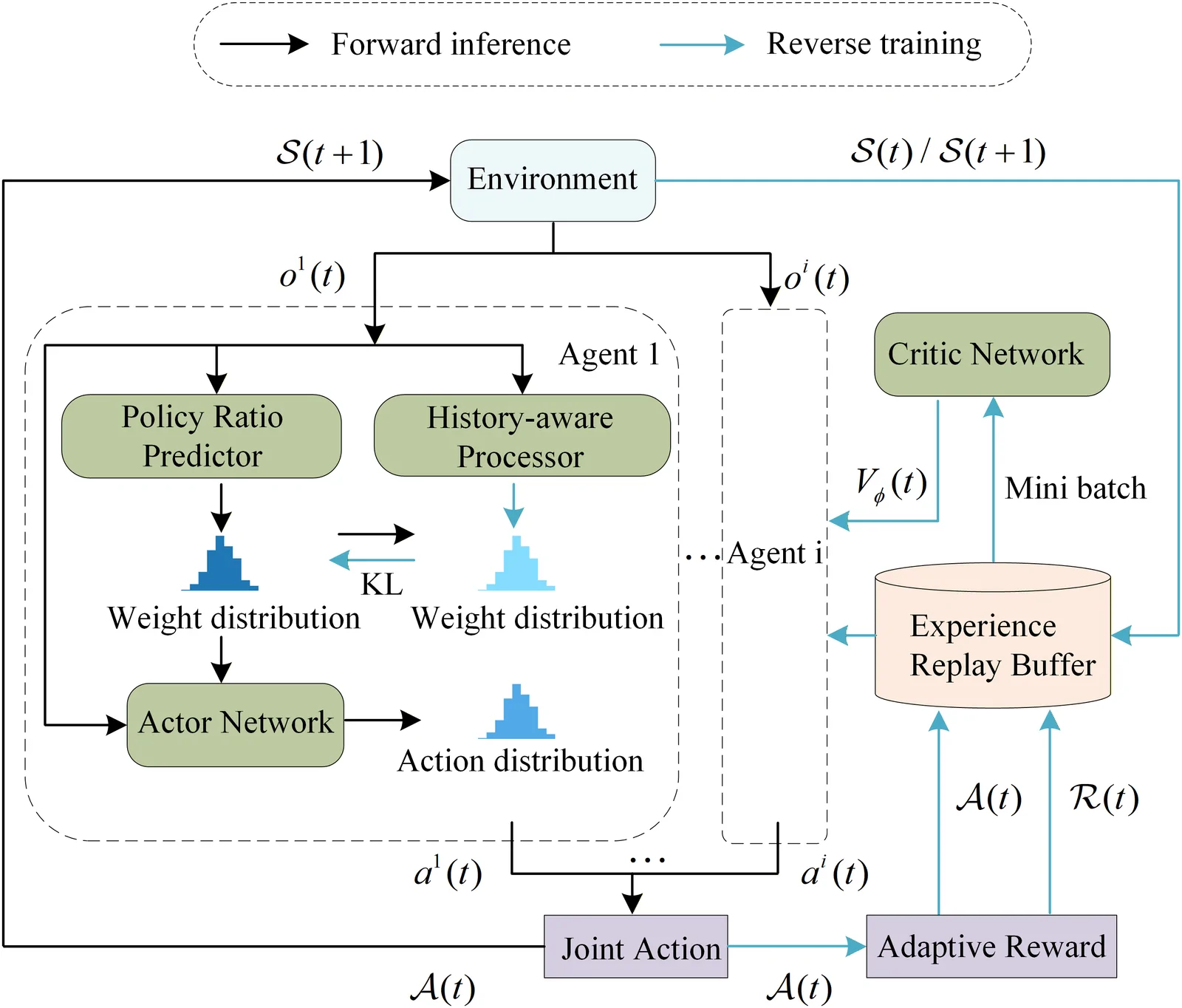
System framework of the proposed W-MAPPO algorithm.

During execution, each agent $u^{i}$ selects actions solely from its local observation $o^{i}(t)$ using two core components: the policy ratio predictor $f_{\theta_{w}^{i}}$, which maps $o^{i}(t)$ to a weight distribution $W_{\theta }^{i}(t)$ over subtasks (detection, allocation, and interception), and the action network $f_{\theta_{a}^{i}}$, which outputs the action $a^{i}(t)$ conditioned on both $o^{i}(t)$ and $W_{\theta }^{i}(t)$. During training, global state information is leveraged through two centralized components: the history-aware processor $f_{\psi^{i}}$, which extracts temporal patterns from historical trajectories to refine policy and value estimates, and the centralized value critic $f_{\phi }$, which estimates the state-value function $V_{\phi }(s(t))$ using the global state s(t)∈𝒮.

### Network architecture design

#### Policy ratio predictor.

The policy ratio predictor $f_{\theta_{w}^{i}}$ is a three-layer fully connected network. It takes the local observation $o^{i}(t)\in {\mathbb{R}}^{\text{Dim}(𝒪)}$ as input and produces the weight distribution:


$$\begin{array}{c} W_{\theta }^{i}(t)=[w_{\text{det}}^{i}(t),\;w_{\text{ass}}^{i}(t),\;w_{\text{int}}^{i}(t)]\in {\mathbb{R}}^{3}, \end{array}$$ (35)

which is normalized by a softmax activation to ensure nonnegativity and sum-to-one constraints:


$$\begin{array}{c} w_{\text{det}}^{i}(t)\geq 0,\;w_{\text{ass}}^{i}(t)\geq 0,\;w_{\text{int}}^{i}(t)\geq 0,\;\sum w_{\cdot }^{i}(t)=1. \end{array}$$ (36)

The vector $W_{\theta }^{i}(t)$ encodes agent $u^{i}$’s attention allocation among the three subtasks.

Although the predictor consumes only the agent’s own observation, inter-agent coordination enters through two channels. First, $o^{i}(t)$ embeds the states and detection information of up to $K_{\max}$ neighbors within *R*_comm_ together with the Voronoi zone encoding, so the generated weights are conditioned on the local multi-agent context. Second, allocation conflicts are explicitly penalized by the distributed competition penalty in Eq. (27), which discourages neighboring agents from concentrating on the same target. Explicit exchange of raw weight vectors among neighboring agents, which would add only three observation dimensions, is identified as a natural lightweight extension in the Limitations section.

#### Action network.

The action network $f_{\theta_{a}^{i}}$ adopts a dual-head architecture with shared hidden layers. The local observation and policy weight distribution are concatenated as input:


$$\begin{array}{c} x^{i}(t)=[o^{i}(t),\;W_{\theta }^{i}(t)]\in {\mathbb{R}}^{\text{Dim}(𝒪)+3}. \end{array}$$ (37)

A fully connected layer with rectified linear unit activation then produces the hidden representation:


$$\begin{array}{c} h^{i}(t)=\mathrm{ReLU}(W_{1}\;x^{i}(t)+b_{1})\in {\mathbb{R}}^{\text{Dim}(h)}, \end{array}$$ (38)

where *W*_1_ and *b*_1_ are respectively the weight matrix and bias vector of the hidden layer and Dim(*h*) is its dimension.

The hidden representation $h^{i}(t)$ is fed into two task-specific output heads:

- The target assignment head outputs a probability distribution over targets:

$$\begin{array}{c} p_{\text{target}}^{i}(t)=\mathrm{softmax}(W_{T}\;h^{i}(t)+b_{T})\in {\mathbb{R}}^{N^{*}}, \end{array}$$ (39)

where $N^{*}$ is the maximum number of planning targets. The task assignment command $C^{i}(t)$ is obtained through discrete sampling from this distribution.

- The motion control head outputs normalized motion commands:

$$\begin{array}{c} {}[v_{c}^{i}(t),\;\theta_{c}^{i}(t)]=\tanh(W_{M}\;h^{i}(t)+b_{M})\in {\mathbb{R}}^{2}. \end{array}$$ (40)

The final executed action is


$$\begin{array}{c} a^{i}(t)=[C^{i}(t),\;v_{c}^{i}(t),\;\theta_{c}^{i}(t)]. \end{array}$$ (41)

This shared representation ensures that target selection and motion control are grounded in a consistent understanding of the environment, improving parameter efficiency.

#### History-aware processor.

The history-aware processor $f_{\psi^{i}}$ is an LSTM-based sequence model that infers the policy ratio weight estimate ${\tilde{W}}_{\theta }^{i}(t)$ and the state-value estimate ${\tilde{V}}_{\phi }^{i}(t)$ from the agent’s historical interaction trajectories.

The network follows an encoder–decoder architecture. The input is the trajectory of the past *H* time steps:


$$\begin{array}{c} \tau^{i}(t)=[x^{i}(t-1),x^{i}(t-2),...,x^{i}(t-H)], \end{array}$$ (42)

where each frame is


$$\begin{array}{c} x^{i}(t-k)=[o^{i}(t-k),a^{i}(t-k),R^{i}(t-k)]\in {\mathbb{R}}^{\text{Dim}(𝒪)+\text{Dim}(𝒜)+1}. \end{array}$$ (43)

The historical trajectory is encoded using a two-layer stacked LSTM with reverse chronological order (i.e., *k* = 1 corresponds to the most recent time step t−1):


$$\begin{array}{cc} h_{k}^{\left(1\right)},c_{k}^{\left(1\right)} & ={\mathrm{LSTM}}^{\left(1\right)}(x^{i}(t-k),h_{k-1}^{\left(1\right)},c_{k-1}^{\left(1\right)}),\;k=1,...,H, \end{array}$$ (44)

$$\begin{array}{cc} h_{k}^{\left(2\right)},c_{k}^{\left(2\right)} & ={\mathrm{LSTM}}^{\left(2\right)}(h_{k}^{\left(1\right)},h_{k-1}^{\left(2\right)},c_{k-1}^{\left(2\right)}),\;k=1,...,H, \end{array}$$ (45)

where $h_{k}^{\left(l\right)}$ and $c_{k}^{\left(l\right)}\in {\mathbb{R}}^{\text{Dim}(h)}$ are the hidden and cell states of the *l*th LSTM layer at step *k*, respectively. The final hidden states from the second *l*ayer form the sequence


$$\begin{array}{c} H_{h}=[h_{1}^{\left(2\right)},h_{2}^{\left(2\right)},...,h_{H}^{\left(2\right)}]\in {\mathbb{R}}^{H\times \text{Dim}(h)}. \end{array}$$ (46)

An attention mechanism is applied to aggregate the *H* hidden states. For each $h_{k}^{\left(2\right)}$, linear projections produce the query $q_{k}$, key $k_{k}$, and value $v_{k}$ vectors. Attention weights are computed as


$$\begin{array}{c} \alpha_{k}=\mathrm{softmax}(q_{k}^{⊤}k_{k}/\sqrt{\text{Dim}(h)}), \end{array}$$ (47)

and the aggregated representation is


$$\begin{array}{c} z=\sum_{k=1}^{H}\alpha_{k}\;v_{k}. \end{array}$$ (48)

Two separate fully connected branches then output the following desired estimates:


$$\begin{array}{cc} {\tilde{W}}_{\theta }^{i}(t) & =\mathrm{softmax}(W_{w}\;z+b_{w})\in {\mathbb{R}}^{3}, \end{array}$$ (49)

$$\begin{array}{cc} {\tilde{V}}_{\phi }^{i}(t) & =W_{v}\;z+b_{v}\in \mathbb{R}, \end{array}$$ (50)

with $W_{w}\in {\mathbb{R}}^{3\times \text{Dim}(h)}$, $b_{w}\in {\mathbb{R}}^{3}$, $W_{v}\in {\mathbb{R}}^{\text{Dim}(h)}$, and $b_{v}\in \mathbb{R}$.

During training, ${\tilde{W}}_{\theta }^{i}(t)$ is used to compute a KL-divergence loss:


$$\begin{array}{c} {ℒ}_{\text{ratio}}=D_{\text{KL}}(W_{\theta }^{i}(t)\;\|\;{\tilde{W}}_{\theta }^{i}(t)), \end{array}$$ (51)

which encourages consistency between the current policy output and the historically inferred weights. Similarly, the value estimate ${\tilde{V}}_{\phi }^{i}(t)$ is used for value regression:


$$\begin{array}{c} {ℒ}_{\text{value}}=({\tilde{V}}_{\phi }^{i}(t)-V_{\phi }^{i}(t){)}^{2}, \end{array}$$ (52)

which distills global knowledge from the centralized critic into the history network. The total loss for the history-aware processor is


$$\begin{array}{c} {ℒ}_{\psi^{i}}={ℒ}_{\text{ratio}}+\lambda_{v}\;{ℒ}_{\text{value}}, \end{array}$$ (53)

where $\lambda_{v}$ is a balancing coefficient.

The attention aggregation in Eq. (47) provides soft temporal adaptation within the fixed window *H*: frames relevant to the current maneuver regime receive higher attention weights, while redundant or stale frames are down-weighted, so the effective receptive field adapts to target behavior even though the window length itself is fixed. The window length *H* is treated as a training hyperparameter, and the sensitivity study in Table 8 shows that performance remains stable across H∈{10,20,30,40}. Fully adaptive horizon selection conditioned on the estimated target maneuver intensity is deferred to future work.

#### Centralized value critic.

The centralized critic $f_{\phi }$ leverages global state information during training to estimate the state-value function. It takes the global state *s*(*t*) as input and outputs:


$$\begin{array}{c} V_{\phi }(t)=[V_{\phi }^{1}(t),V_{\phi }^{2}(t),...,V_{\phi }^{M}(t){]}^{⊤}\in {\mathbb{R}}^{M}. \end{array}$$ (54)

The network comprises a shared feature extractor followed by agent-specific value heads, trained by minimizing the temporal-difference error:


$$\begin{array}{c} {ℒ}_{\phi }=𝔼\left[(r(t)+\gamma V_{\phi }(t+1)-V_{\phi }(t){)}^{2}\right]. \end{array}$$ (55)

This module is used only during centralized training to compute advantage estimates and provide supervision signals to the history-aware processor.

## Algorithm training procedure

The training procedure follows the CTDE paradigm (Algorithm 1). Each episode alternates between a decentralized execution phase (lines 10–17), where agents interact with the environment using only local observations, and a centralized training phase (lines 19–29), where global information is exploited to update all network parameters.

**Algorithm 1 W-MAPPO**

**Require:** Environment env, number of agents *M*, number of threat targets *N*, maximum steps *T*

**Ensure:** Trained policy network

1: **Initialization:** (all agents share parameters $\theta_{w}$, $\theta_{a}$, ψ)

2: Policy ratio predictors $\{f_{\theta_{w}}^{i}{\}}_{i=1}^{M}$

3: Action networks $\{f_{\theta_{a}}^{i}{\}}_{i=1}^{M}$

4: History-aware processors $\{f_{\psi }^{i}{\}}_{i=1}^{M}$

5: Centralized value critic $f_{\phi }$

6: Experience replay buffer 𝒟←∅

7: **for** Episode = 1 **to** MaxEpisodes **do**

8: Initialize environment, obtain initial state *s*(0) and observations $\left\{o^{i}(0)\right\}$

9: **for** *t* = 0 **to** *T* **do**

10: **Decentralized execution phase:**

11: $W_{\theta }^{i}(t)\leftarrow f_{\theta_{w}^{i}}(o^{i}(t))$

12: $a^{i}(t)\leftarrow f_{\theta_{a}^{i}}(o^{i}(t),W_{\theta }^{i}(t))$

13: Record old policy probability $\pi_{\text{old}}^{i}(t)$

14: Execute joint action $a(t)\leftarrow [a^{1}(t),...,a^{M}(t)]$

15: Environment step: $\left\{s(t+1)\},\{o^{i}(t+1)\},r(t)\leftarrow \text{env.step}(a(t)\right)$

16: Store experience tuple in 𝒟

17: **end for**

18: **if** |𝒟|≥BatchSize **then**

19: **Centralized training phase:**

20: **for** epoch = 1 **to** *k* **do**

21: batch ←𝒟.sample(BatchSize)

22: Update $f_{\phi }$ by minimizing ${ℒ}_{\phi }^{\text{critic}}$ (see Eq. 56)

23: Compute advantage: $A_{\theta_{a}}(t)\leftarrow \mathrm{GAE}(V_{\phi }(t),r(\cdot ))$

24: Extract historical trajectory $\tau^{i}(t)$

25: ${\tilde{W}}_{\theta }^{i}(t),{\tilde{V}}_{\phi }^{i}(t)\leftarrow f_{\psi^{i}}(\tau^{i}(t))$

26: Update $\{f_{\theta_{a}^{i}}{\}}_{i=1}^{M}$ by minimizing ${ℒ}_{\theta_{a}^{i}}^{\text{actor}}$ (see Eq. 57)

27: Update $\{f_{\theta_{w}^{i}}{\}}_{i=1}^{M}$ jointly with the policy network

28: Update $\{f_{\psi^{i}}{\}}_{i=1}^{M}$ by minimizing ${ℒ}_{\psi^{i}}$ (see Eq. 53)

29: **end for**

30: **end if**

31: **end for**

During centralized training, the critic is updated by minimizing the temporal-difference error:


$$\begin{array}{c} {ℒ}_{\phi }^{\text{critic}}=\frac{1}{N_{\text{batch}}}\sum_{b=1}^{N_{\text{batch}}}{\left(r(t)+\gamma V_{\phi }(t+1)-V_{\phi }(t)\right)}^{2}, \end{array}$$ (56)

where γ is the discount factor. The advantage function $A_{\theta_{a}}(t)$ is computed via generalized advantage estimation (GAE) [40].

The policy network is updated using the PPO-clip objective. Let the importance sampling ratio be $\rho_{\theta_{a}}^{i}(t)=\pi_{\theta_{a}}^{i}(a^{i}(t)|o^{i}(t))/\pi_{\theta_{a}^{\text{old}}}^{i}(a^{i}(t)|o^{i}(t))$. The total actor loss combines the clipped surrogate, entropy regularization, and historical consistency:


$$\begin{array}{c} {ℒ}_{\theta_{a}^{i}}^{\text{actor}}=\underset{\text{PPO-clip objective}}{\underbrace{𝔼\left[\min\left(\rho_{\theta_{a}}^{i}A_{\theta_{a}},\mathrm{clip}\left(\rho_{\theta_{a}}^{i},1-\varepsilon ,1+\varepsilon \right)A_{\theta_{a}}\right)\right]}}-\lambda_{\text{ent}}ℋ(\pi_{\theta_{a}}^{i})+\lambda_{\text{hist}}\mathrm{KL}\left(W_{\theta }^{i}\;\|\;{\tilde{W}}_{\theta }^{i}\right), \end{array}$$ (57)

where ε is the clipping threshold, $\lambda_{\text{ent}}$ controls exploration via entropy bonus, and $\lambda_{\text{hist}}$ enforces temporal smoothness of the dynamic weights by penalizing divergence from the history-aware processor’s prediction ${\tilde{W}}_{\theta }^{i}$. The history-aware processor $f_{\psi^{i}}$ is updated by minimizing Eq. (53), and the policy ratio predictor $f_{\theta_{w}^{i}}$ is updated jointly with the action network through the actor loss gradient.

### Training stability characterization

To complement the empirical evaluation, we provide a concise stability characterization of W-MAPPO under standard boundedness, smoothness, and sufficient-sampling assumptions. This analysis is intended to clarify why the dynamic weighting mechanism remains trainable under policy-gradient optimization, rather than to claim global optimality. The dynamic weight mechanism introduces a time-varying composite reward


$$\begin{array}{c} R^{i}(t)=\sum_{j\in \{\text{det},\text{ass},\text{int}\}}w_{j}^{i}(t)R_{j}^{i}(t), \end{array}$$ (58)

which may induce reward non-stationarity. The following results summarize how parameter sharing, PPO-style bounded policy updates, and historical-consistency regularization jointly limit this effect.

**Lemma 1 (Shared-policy aggregation under parameter sharing).** *In W-MAPPO, all M agents share the policy parameters* $\theta =(\theta_{a},\theta_{w})$*, where* $\theta_{a}$ *denotes the action-network parameters and* $\theta_{w}$ *denotes the policy-ratio predictor parameters. Defining the population objective as*


$$\begin{array}{c} \bar{J}(\theta )=\frac{1}{M}\sum_{i=1}^{M}J^{i}(\theta ),\;J^{i}(\theta )={𝔼}_{\tau^{i}\sim \pi_{\theta }}\left[\sum_{t=0}^{T}\gamma^{t}R^{i}(t)\right], \end{array}$$ (59)

*the aggregated multi-agent update estimates the gradient of* $\bar{J}(\theta )$:


$$\begin{array}{c} \nabla_{\theta }\bar{J}(\theta )=\frac{1}{M}\sum_{i=1}^{M}\nabla_{\theta }J^{i}(\theta ). \end{array}$$ (60)

*If the per-agent gradient estimators have variance bounded by* $\sigma^{2}$*, the averaged estimator has variance at most* $\sigma^{2}/M$ *under independent rollouts.*

*Proof.* The result follows from linearity of expectation and the standard policy-gradient identity. Since all agents share the same policy and their rollout batches are aggregated in the shared buffer, averaging the *M* per-agent gradient estimators yields an unbiased estimator of the population gradient. Under independent rollouts, the variance of the average is reduced by a factor of *M*. □

**Lemma 2 (Approximate bounded divergence under PPO-style clipping).** *Let* $\pi_{\theta^{\left(k\right)}}$ *and* $\pi_{\theta^{\left(k+1\right)}}$ *denote consecutive policies. If the effective post-update importance ratio remains within* [1−ε,1+ε] *on the sampled state-action support and the rollout batch sufficiently covers the effective support, then the KL divergence between consecutive policies is approximately bounded by*


$$\begin{array}{c} D_{\text{KL}}(\pi_{\theta^{\left(k\right)}}\;\|\;\pi_{\theta^{\left(k+1\right)}})\leq -\log(1-\varepsilon )\leq \varepsilon +\varepsilon^{2}, \end{array}$$ (61)

*for* ε∈(0,0.5].

*Proof.* For the importance ratio $\rho (s,a)=\pi_{\theta^{\left(k+1\right)}}(a|s)/\pi_{\theta^{\left(k\right)}}(a|s)$, the assumed effective post-update ratio bound gives ρ(s,a)≥1−ε on the sampled effective support. Therefore, −logρ(s,a)≤−log(1−ε). Taking the expectation over actions gives the stated approximate KL bound, and the second inequality follows from the Taylor expansion of −log(1−ε). □

**Theorem 1 (Approximate stationary-region characterization).** *Assume that: (C1) subtask rewards are bounded by* $R_{\max}$*; (C2) dynamic weights lie on the probability simplex; (C3) the dynamic-weight drift is bounded under historical-consistency regularization as* $\|W_{\theta }^{i,(k+1)}-W_{\theta }^{i,(k)}{\|}_{1}\leq \sqrt{2\delta_{KL}}$*; (C4) the population objective has an L-Lipschitz continuous gradient; (C5) stochastic gradient variance is bounded by* $\sigma^{2}/M$*; and (C6) the learning-rate schedule satisfies the standard Robbins-Monro conditions. Then the iterates approach an approximate stationary region satisfying*


$$\begin{array}{c} \frac{1}{K}\sum_{k=0}^{K-1}𝔼\left[{\left\|\nabla_{\theta }\bar{J}(\theta^{\left(k\right)})\right\|}^{2}\right]\leq \frac{2[\bar{J}(\theta^{*})-\bar{J}(\theta^{\left(0\right)})]}{K\bar{\alpha }}+\frac{L\sigma^{2}\bar{\alpha }}{M}+2C_{b}^{2}\epsilon_{R}^{2}, \end{array}$$ (62)

*where* $\epsilon_{R}=R_{\max}\sqrt{2\delta_{KL}}$, $C_{b}=2\gamma /(1-\gamma {)}^{2}$, $\bar{\alpha }=K^{-1}\sum_{k=0}^{K-1}\alpha_{k}$*, and* $\theta^{*}$ *is a maximizer of* $\bar{J}$*. Consequently,*


$$\begin{array}{c} \underset{K\to \infty }{lim inf}\frac{1}{K}\sum_{k=0}^{K-1}𝔼\left[{\left\|\nabla_{\theta }\bar{J}(\theta^{\left(k\right)})\right\|}^{2}\right]\leq 𝒪\;\left(R_{\max}^{2}\delta_{KL}\right). \end{array}$$ (63)

*Proof.* The proof follows standard stochastic policy-gradient arguments. Conditions (C1) and (C2) bound the composite reward and the corresponding value and advantage terms. Condition (C3), together with Pinsker’s inequality, bounds the reward drift caused by changes in the dynamic weights as $\epsilon_{R}=R_{\max}\sqrt{2\delta_{KL}}$. This reward drift introduces a bounded gradient bias proportional to $C_{b}\epsilon_{R}$. Applying the smoothness inequality to the stochastic update, using the variance reduction in Lemma 1, and summing the resulting bound over *K* iterations gives Eq. (62). Taking K→∞ under the learning-rate condition yields Eq. (63).

This result should be interpreted as a stability characterization rather than a global optimality guarantee. It indicates that, when dynamic-weight drift is controlled by historical-consistency regularization, W-MAPPO remains within an approximate stationary region whose residual size is governed by the bounded reward drift.

## Simulation experiments

This section validates the proposed W-MAPPO algorithm through comprehensive simulation experiments. We first describe the environment setup and evaluation metrics, then present comparative results against representative baselines, followed by ablation studies and a case study demonstrating the algorithm’s behavior in representative interception scenarios.

### Experimental setup

#### Environment setup of the maneuvering interception task.

(1) Initial Environment Setup

The maneuvering interception environment comprises two components: the multi-agent defense system and the threat target cluster. During initialization, agents are uniformly generated along the circumference of radius *R*_form_ around *Z*_core_, with random perturbations *R*_jitter_ applied to their positions and random initial headings.

Threat targets are randomly generated outside *Z*_task_: an azimuth angle is sampled from [0,2π], a base anchor point is determined at radius *R*_linear_, and a perturbation *R*_jitter_ is applied. Initial headings point toward the coordinate origin, simulating centripetal attack intent.

This initialization ensures both structural stability and tactical rationality of the defense formation, while random perturbations enhance training sample diversity, enabling agents to develop generalizable strategies.

(2) Threat Target Maneuvering Model

To simulate the intelligence and uncertainty of threat targets, $\pi_{w}$ is modeled as a hybrid maneuvering model with four modes: (1) *Directed invasion*: baseline behavior moving toward *Z*_core_ at constant speed *v*_nor_; (2) *Random walk*: heading modulated by Gaussian noise $\omega_{w}^{j}\sim N(0,\sigma_{\omega }^{2})$ with transition probability *p*_turn_; (3) *Pulse acceleration*: intermittent high-speed penetration (acceleration → hold → deceleration) with probability *p*_acc_; (4) *Feedback evasion*: when detected, targets evade away from the nearest agent with probability *p*_avoid_. The feedback evasion mode has the highest priority and can override other modes. Each mode is maintained for a minimum duration $\Delta T_{\text{mode}}$ to ensure trajectory continuity. These four modes collectively span the adversarial behavior spectrum from non-reactive to actively adversarial, ensuring that the trained policy is evaluated against diverse threat intelligence levels.

(3) Experimental Evaluation Metrics

Consistent with the problem formulation in the System Modeling and Problem Formulation section, particularly the comprehensive defense performance metric in Eq. (5), the evaluation focuses on interception effectiveness, protection effectiveness, defense depth, and operational efficiency. Specifically, the task success rate and average target interception rate correspond to the breach-prevention and interception terms in Eq. (5), the average defense radius reflects the defense-depth term, and the average interception time is additionally reported to assess response efficiency under varying threat-density scenarios. The following metrics are employed:

- **Task success rate:** Interception is considered successful if all threat targets are intercepted outside *Z*_core_. Otherwise, if any threat target enters *Z*_core_, the task is deemed to have failed. The task success rate is thus defined as the proportion of successful trials across multiple experiments.
- **Average target interception rate:** When the task fails, the ratio of intercepted threat targets to the total number of targets when a threat target enters *Z*_core_ is the target interception rate for that experiment. When the task succeeds, the target interception rate is 100%. The average target interception rate is the mean across multiple trials.
- **Average interception time:** In a single experiment, the average time steps when each threat target is intercepted is recorded as the interception time. The average interception time is the mean across multiple trials.
- **Average defense radius:** In a single experiment, the average distance from the region center (coordinate origin) when each threat target is intercepted is recorded as the defense radius. The average defense radius is the mean across multiple trials.

#### Experimental environment and parameter settings.

Experiments are conducted on a platform with an Intel i9-14900KF CPU, NVIDIA RTX 4090 GPU, and 64 GB RAM. PyTorch serves as the deep learning framework, and OpenAI Gym provides the reinforcement learning interface.

The experimental conditions are:

1. All agents are homogeneous with identical performance parameters, and a parameter-sharing strategy is used to facilitate parallel learning during training. This design ensures that the learned policy is permutation-invariant with respect to agent identities, enabling direct deployment to different team sizes without retraining. To stress-test coordination capabilities under resource constraints, experiments fix the agent count at 5 while varying threat density (5 vs 5, 5 vs 10, 5 vs 15), progressively intensifying detection-interception competition.
2. All agent decisions are completely end-to-end, with no explicit specification of training phases. The reinforcement learning framework automatically learns task phase switching through rewards.
3. The simulation environment parameters are dimensionless but scaled according to real physical environments, facilitating future transfer to physical platforms. The main parameters are shown in Table 2.
4. The reward function parameters are determined through multiple trials and parameter adjustments, as shown in Table 3.
5. The algorithm training hyperparameters are shown in Table 4.

**Table 2:** Task environment parameter settings.

Parameter Name	Symbol	Value
Number of agents	*M*	5
Number of threat targets	*N*	5–15
Agent initial formation radius	*R*_form_	6
Threat target linear radius	*R*_linear_	18
Position random perturbation	*R*_jitter_	2
Task area horizontal range	$L_{x}$	20
Task area vertical range	$L_{y}$	20
Core area radius	*R*_core_	1
Communication radius	*R*_comm_	6
Detection radius	*R*_detect_	3
Interception radius	*R*_int_	2
Agent maximum speed	$v_{c}^{\max}$	1.0
Agent minimum speed	$v_{c}^{\min}$	0
Agent maximum linear acceleration	$a_{c}^{\max}$	0.3
Agent maximum angular velocity	$\omega_{U}^{\max}$	1
Agent maximum lateral acceleration	$a_{\max}$	0.8
Random walk mode probability	*p*_turn_	25%
Random walk angular velocity std.	$\sigma_{\omega }$	0.2
Random pulse acceleration probability	*p*_acc_	25%
Feedback evasion probability	*p*_avoid_	80%
Feedback evasion gain	$\alpha_{\text{escape}}$	1.047
Mode duration	$\Delta T_{\text{mode}}$	3.0
Threat target baseline speed	*v*_nor_	0.6
Threat target maximum speed	$v_{w}^{\max}$	0.8
Threat target acceleration	$a_{w}$	0.15
Threat target deceleration	$-a_{w}$	0.15
High-speed holding duration	*T*_hold_	3.0
Threat target maximum steering angular velocity	$\omega_{T}^{\max}$	0.5
Agent neighbor count	$K_{\max}$	4

**Table 3:** Reward function parameter settings.

Parameter Name	Symbol	Value
**Collaborative detection reward $R_{\text{det}}^{i}(t)$**		
New target discovery reward coefficient	$\alpha_{\text{det}}$	10
Area exploration reward coefficient	$\alpha_{\text{exp}}$	5
Initial exploration reward adjustment coefficient	$\beta_{\text{exp}}$	2
Voronoi responsibility area collaborative coefficient	$\alpha_{\text{vor}}$	2
Sampling point count	$N_{s}$	50
**Task assignment reward $R_{\text{ass}}^{i}(t)$**		
Distance matching reward reference distance	*d*_ref_	5
Distance matching reward scaling coefficient	$\alpha_{\text{dis}}$	1
Competition penalty scaling coefficient	$\beta_{\text{comp}}$	5
Task switching penalty scaling coefficient	$\beta_{\text{switch}}$	3
**Interception reward $R_{\text{int}}^{i}(t)$**		
Approach reward scaling coefficient	$\alpha_{\text{appr}}$	2
Angle advantage reward scaling coefficient	$\alpha_{\text{angle}}$	1.5
Successful interception reward coefficient	$\alpha_{\text{inter}}$	30

**Table 4:** Training hyperparameter settings.

Parameter Name	Symbol	Value
Number of parallel environments	*N*_env_	16
Single cycle steps	$T_{\max}$	300
Single sampling steps	*L*_rollout_	200
Training batch size	*B*_batch_	6000
Minibatch size	*B*_minibatch_	1200
Stochastic gradient descent iteration rounds	*K*_epochs_	10
Learning rate	$l_{r}$	0.0002
Discount factor	γ	0.98
GAE advantage estimation parameter	λ	0.95
LSTM hidden layer dimension	Dim(*h*)	64
LSTM time step length	*H*	20
Maximum detectable targets per agent	$N^{\prime }$	5
Maximum planning targets per agent	$N^{*}$	20
Policy ratio predictor hidden dimensions	$d_{1},d_{2}$	128, 64
Centralized critic global state dimension	$D_{s}$	200
Centralized critic hidden dimension	$d_{c}$	128
PPO clip threshold	ε	0.2
Entropy regularization coefficient	$\lambda_{\text{ent}}$	0.01
KL regularization coefficient	$\lambda_{\text{hist}}$	0.05

### Comparative experimental analysis

To comprehensively evaluate the performance of the proposed W-MAPPO method, we compare it against two categories of baselines: classical optimization pipelines that do not require training, and MARL methods trained under identical conditions. All methods operate under the same partially observable, decentralized execution setting as W-MAPPO, ensuring a fair comparison.

**Classical baselines (no training required):**
  - *Local Greedy + PN* (Proportional Navigation): Each agent independently selects the nearest detected target within *R*_detect_ and pursues it using the proportional navigation guidance law with navigation constant *N*_PN_ = 3. When no target is detected, the agent executes a random patrol within its local region. This baseline represents the simplest rule-based strategy under partial observability, with no inter-agent coordination.
  - *Local Hungarian + PN*: Agents within mutual communication range (*R*_comm_) construct a local cost matrix based on distances to all jointly detected targets and solve a local Hungarian assignment. Each agent then pursues its assigned target using proportional navigation. Undetected targets are not included in the assignment. When no targets are detected by the local group, agents perform Voronoi-based patrol. This baseline represents a classical optimization approach with local coordination under partial observability.
**MARL baselines:**
  - *MAPPO* [41]: The standard Multi-Agent Proximal Policy Optimization algorithm based on the Actor-Critic framework, serving as our primary MARL baseline. It uses the same network dimensions and training hyperparameters as W-MAPPO but lacks the dynamic weight mechanism and history-aware processor.
  - *IPPO* [42] (Independent PPO): Each agent trains an independent PPO policy using only its local observations, without parameter sharing or centralized critic. This method represents the fully decentralized learning baseline.
  - *MADDPG* [43] (Multi-Agent Deep Deterministic Policy Gradient): A continuous-action MARL algorithm that employs centralized critics with decentralized actors. Each agent’s critic has access to the observations and actions of all agents during training, while execution relies solely on local observations.
  - *QPLEX* [44] (QPLEX: Duplex Dueling Multi-Agent Q-Learning): A value decomposition method that achieves full expressiveness of the joint action-value function through a duplex dueling architecture, overcoming the monotonicity constraint of QMIX. To accommodate the hybrid action space, continuous motion control actions are discretized into a finite set of heading and speed combinations.
  - *MAT* [45] (Multi-Agent Transformer): A Transformer-based cooperative MARL method that models the joint policy as a sequence generation problem. It leverages multi-head attention to capture inter-agent dependencies and generates actions sequentially, enabling expressive coordination without explicit communication protocols.
  - *DASF-GRL* [36] (Dynamic Agent-Scaling Framework with Game-augmented Reinforcement Learning): A recent method specifically designed for defensive counter-air operations. It dynamically adjusts the number of active defenders based on real-time threat levels and incorporates a safety barrier function from differential game theory to constrain action spaces. We adapt it to our fixed-agent-count setting by disabling the scaling mechanism while retaining the game-augmented reward shaping and barrier constraints.
  - *MRPM* [35] (Modified Region Protection Method): A hybrid evolutionary reinforcement learning approach that combines differential evolution (DE) with MADDPG for cooperative region protection. It maintains a population of policies and uses evolutionary operators to escape local optima, while MADDPG provides gradient-based fine-tuning within each generation.

The specific experimental settings are as follows: the two classical baselines use identical kinematic parameters and detection/communication constraints as the learning-based methods, differing only in their decision-making logic. All MARL methods are trained under the same partially observable environment with identical observation and action spaces. MAPPO and IPPO use the same PPO-based policy architecture, with MAPPO employing a centralized critic and IPPO using independent local critics. MADDPG employs continuous action outputs with centralized critics that observe joint states during training. QPLEX uses the duplex dueling architecture with discretized motion control actions (9 heading directions × 3 speed levels). MAT uses a 2-layer Transformer encoder-decoder with 4 attention heads and hidden dimension 64, matching the parameter scale of other methods. DASF-GRL uses its original game-augmented reward structure and safety barrier constraints, adapted to the fixed 5-agent setting. MRPM maintains a population of 10 MADDPG-based policies with DE-based crossover and mutation operators. All MARL methods share the same training budget of $1.5\times 10^{6}$ time steps. All methods (including classical baselines) are evaluated over 100 independent simulation runs per scenario.

(1) Training Convergence and Sample Efficiency

To illustrate the learning dynamics of each MARL algorithm across different task scales, Fig 5 presents the training reward curves of all eight learning-based algorithms (W-MAPPO plus the seven MARL baselines) in the 5 vs 5, 5 vs 10, and 5 vs 15 scenarios. Classical baselines (Local Greedy + PN and Local Hungarian + PN) are excluded from this comparison as they do not involve a training process. Each curve is averaged over 10 independent runs with different random seeds, and the shaded region indicates ±1 standard deviation.

**Fig 5:**
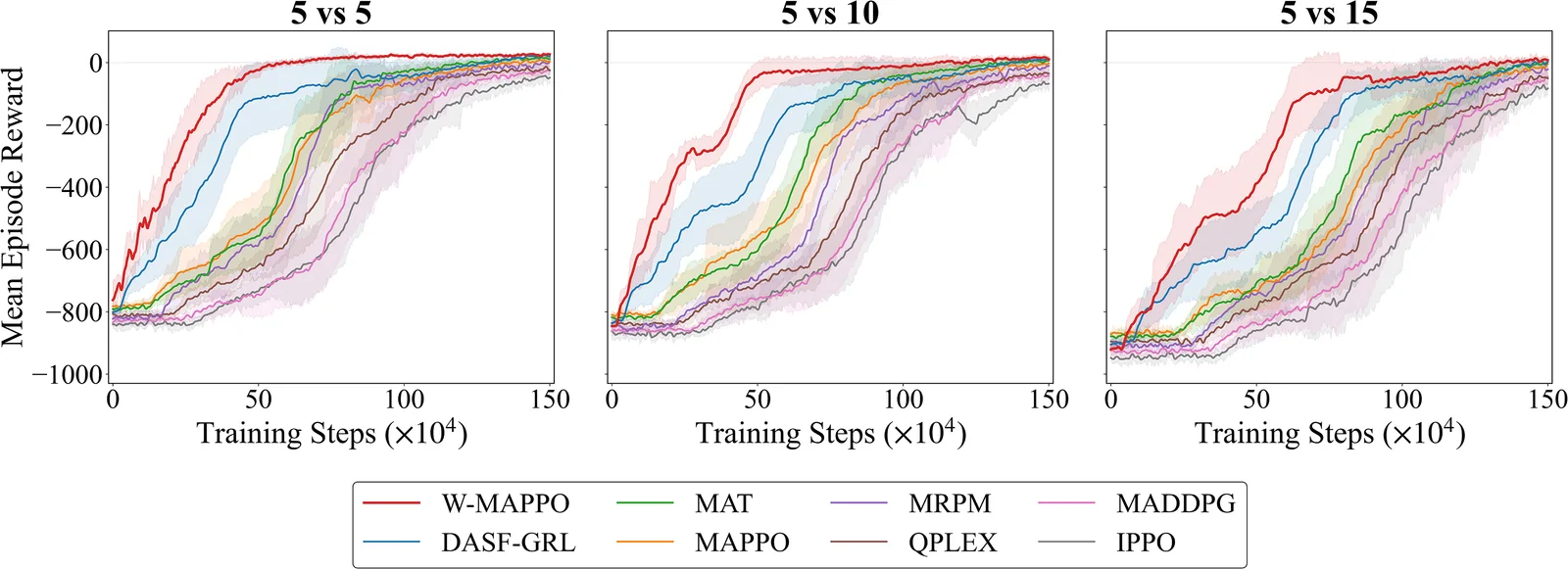
Training reward curves of all algorithms across three scenarios: 5 vs 5 (left), 5 vs 10 (middle), and 5 vs 15 (right). Each curve is averaged over 10 seeds with ±1 standard deviation shading. The performance ranking remains consistent across scales, while convergence slows and final rewards decrease as the number of threats increases.

Key observations from Fig 5 include: (1) Regarding final performance, a clear and consistent hierarchy emerges across all three scenarios: IPPO < MADDPG < QPLEX < MRPM ≈ MAPPO < MAT < DASF-GRL < W-MAPPO. In the 5 vs 5 scenario, W-MAPPO converges to 24.1±1.1, outperforming the second-best DASF-GRL (17.3±2.2) and standard MAPPO (2.2±3.6, Δ=21.9), validating the effectiveness of the dynamic weight mechanism and history-aware modules; (2) Regarding convergence speed, methods with explicit coordination mechanisms (W-MAPPO, DASF-GRL, MAT) converge within 4.2–$8.2\times 10^{5}$ steps, while methods relying on implicit coordination or independent learning require 8.1–$11.7\times 10^{5}$ steps. DASF-GRL exhibits rapid initial rise due to its game-augmented reward shaping but slows in the mid-training phase, whereas W-MAPPO maintains fast convergence throughout; (3) Regarding performance under increasing threat density, as the number of threats increases from 5 to 15 with the number of agents fixed at 5, all algorithms experience degraded performance and slower convergence, yet the relative ranking remains strictly preserved. W-MAPPO maintains positive rewards even in the most challenging 5 vs 15 scenario (5.4), while weaker methods suffer disproportionate degradation (e.g., IPPO drops from −56.6 to −107.5). The performance gap between W-MAPPO and MAPPO remains stable (Δ≈20–22) across all threat-density settings, confirming that the proposed coordination mechanism provides robust improvement under increasing multi-threat complexity.

(2) Task-Level Performance Comparison

Three scenarios with 5, 10, and 15 threat targets (all involving 5 agents) are evaluated. Performance metrics are aggregated over 100 independent runs, with results presented in Table 5.To provide a more intuitive comparison, Fig 6 visualizes the four key performance metrics across all scenarios.

**Table 5:** Comparative performance of various interception algorithms under different threat-density scenarios.

Algorithm	Success	Avg. Intercept.	Avg. Time	Avg. Radius
**Scenario: 5 vs 5**				
W-MAPPO	**94%**	**95.20%**	**72.34 ± 5.18**	**8.12 ± 1.08**
DASF-GRL	91%	92.40%	78.52 ± 7.36	7.58 ± 1.25
MAT	88%	89.80%	80.67 ± 8.14	7.41 ± 1.33
MAPPO	85%	88.20%	83.18 ± 8.72	7.04 ± 1.45
MRPM	83%	87.40%	81.93 ± 9.28	7.18 ± 1.52
QPLEX	78%	84.60%	88.45 ± 10.63	6.63 ± 1.61
MADDPG	74%	82.80%	91.26 ± 11.47	6.38 ± 1.74
IPPO	70%	79.60%	96.83 ± 13.25	6.05 ± 1.82
Local Hungarian + PN	64%	76.80%	102.57 ± 14.83	5.74 ± 1.93
Local Greedy + PN	56%	71.40%	112.38 ± 18.42	5.21 ± 2.15
**Scenario: 5 vs 10**				
W-MAPPO	**78%**	**84.40%**	**81.47 ± 6.53**	**7.56 ± 0.94**
DASF-GRL	71%	79.20%	86.93 ± 8.72	7.14 ± 1.38
MAT	68%	76.40%	89.28 ± 9.85	6.78 ± 1.47
MAPPO	63%	72.60%	92.64 ± 10.16	6.52 ± 1.56
MRPM	61%	73.80%	90.85 ± 10.52	6.68 ± 1.63
QPLEX	57%	69.20%	97.52 ± 12.08	6.08 ± 1.78
MADDPG	53%	66.80%	101.34 ± 12.94	5.82 ± 1.85
IPPO	49%	63.40%	108.26 ± 15.17	5.47 ± 1.96
Local Hungarian + PN	42%	58.60%	115.82 ± 17.64	5.12 ± 2.04
Local Greedy + PN	33%	51.80%	126.45 ± 21.37	4.58 ± 2.31
**Scenario: 5 vs 15**				
W-MAPPO	**67%**	**79.40%**	**86.52 ± 7.38**	**7.31 ± 1.62**
DASF-GRL	60%	69.80%	91.78 ± 9.43	6.82 ± 1.51
MAT	54%	65.20%	96.43 ± 10.87	6.24 ± 1.58
MAPPO	51%	62.40%	99.15 ± 11.56	6.01 ± 1.67
MRPM	49%	60.60%	97.84 ± 11.93	6.14 ± 1.75
QPLEX	44%	56.80%	105.62 ± 13.84	5.52 ± 1.88
MADDPG	41%	53.40%	109.38 ± 14.62	5.28 ± 1.97
IPPO	36%	50.20%	116.74 ± 17.05	4.93 ± 2.08
Local Hungarian + PN	30%	46.20%	124.36 ± 19.82	4.61 ± 2.18
Local Greedy + PN	22%	39.80%	135.72 ± 23.56	4.12 ± 2.43

**Fig 6:**
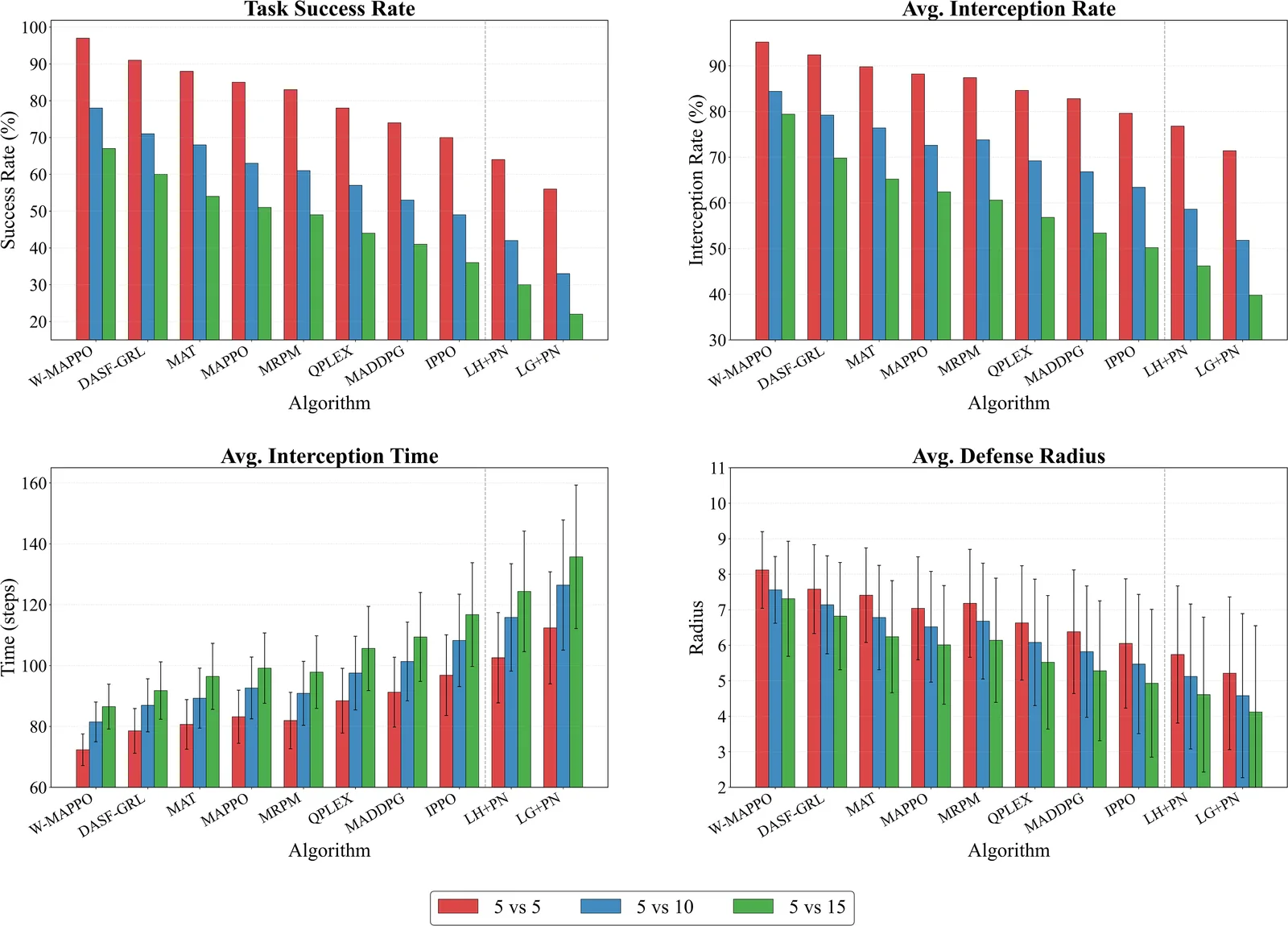
Grouped bar chart comparison of four performance metrics across three threat-density scenarios: task success rate (top-left), average interception rate (top-right), average interception time (bottom-left), and average defense radius (bottom-right). The dashed vertical line separates MARL methods (left) from classical baselines (right). W-MAPPO achieves the best values in all metrics under all threat-density scenarios, and all learning-based methods substantially outperform the classical baselines.

As shown in Table 5 and Fig 6, key findings include: (1) W-MAPPO achieves the highest success rates across all scenarios (94%, 78%, 67%) while maintaining the best time efficiency (72.34, 81.47, 86.52 steps) and the largest defense radii; (2) All learning-based methods substantially outperform the classical baselines, demonstrating the necessity of adaptive coordination. Even the weakest MARL method (IPPO) outperforms Local Hungarian + PN by 6–8% in success rate across all scenarios, confirming that learned policies provide superior detection-interception coordination compared to rule-based strategies; (3) Among classical baselines, Local Hungarian + PN consistently outperforms Local Greedy + PN (e.g., 64% vs. 56% in 5 vs 5), validating that local coordination improves performance even without learning. However, the gap between Local Hungarian + PN and MARL methods widens substantially as threat density increases (from 6% to 14% gap with IPPO), indicating that rule-based coordination struggles in complex multi-threat environments under fixed defensive resources; (4) As the number of threats increases from 5 to 15, all algorithms exhibit degraded performance, yet the relative ranking remains stable. W-MAPPO’s success rate decreases by 27 percentage points (from 94% to 67%), while Local Greedy + PN drops by 34 percentage points (from 56% to 22%), indicating that coordination-based methods degrade more gracefully under increasing multi-threat complexity; (5) The performance gap between the top-tier methods (W-MAPPO, DASF-GRL, MAT) and the remaining algorithms widens substantially under higher threat-density settings, with W-MAPPO maintaining a 9–16% success rate advantage over standard MAPPO across all three scenarios.

Statistical significance is verified using the Wilcoxon signed-rank test on per-episode returns across 100 runs. W-MAPPO significantly outperforms all baselines at *p* < 0.01 in all three scenarios. Among the top-tier methods, the pairwise comparisons W-MAPPO vs. DASF-GRL and W-MAPPO vs. MAT yield *p* < 0.05 in the 5 vs 5 scenario and *p* < 0.01 in the 5 vs 10 and 5 vs 15 scenarios, confirming that the observed performance gaps are statistically meaningful.

(3) Computational Cost

Table 6 reports the computational overhead of each method. W-MAPPO’s per-step inference time (2.8 ms) is marginally higher than MAPPO (1.9 ms) due to the Policy Ratio Predictor and LSTM modules, but remains well within real-time requirements for the control period Δt used in our experiments. The training overhead is moderate: W-MAPPO requires 4.2 hours compared to 3.1 hours for MAPPO, a 35% increase attributable to the history-aware processor’s sequential computation. Classical baselines require no training and have negligible inference cost.

**Table 6:** Computational cost comparison (5 vs 5 scenario, RTX 4090).

Algorithm	Inference	Training (h)	GPU Mem.
W-MAPPO	2.8	4.2	3.8
DASF-GRL	3.1	5.6	4.2
MAT	4.5	6.8	5.1
MAPPO	1.9	3.1	2.6
MRPM	6.2	9.4	4.8
QPLEX	2.4	4.8	3.4
MADDPG	2.1	3.8	3.1
IPPO	1.7	2.6	2.2
Local Hungarian + PN	0.3	–	–
Local Greedy + PN	0.1	–	–

### Ablation study

To verify the necessity and effectiveness of each key module, we conducted 100 repeated ablation experiments in the 5 vs 5 scenario, systematically removing or varying the dynamic-weight module, history-aware module, and Voronoi detection mechanism.

(1) Validation of the dynamic-weight module

Fig 7 shows the real-time weight trajectories for a single agent during an episode. The weights adapt to task context: path planning dominates upon target detection, task allocation increases during target matching, and collaborative detection rises after threats are eliminated, confirming the mechanism’s adaptive capability.

**Fig 7:**
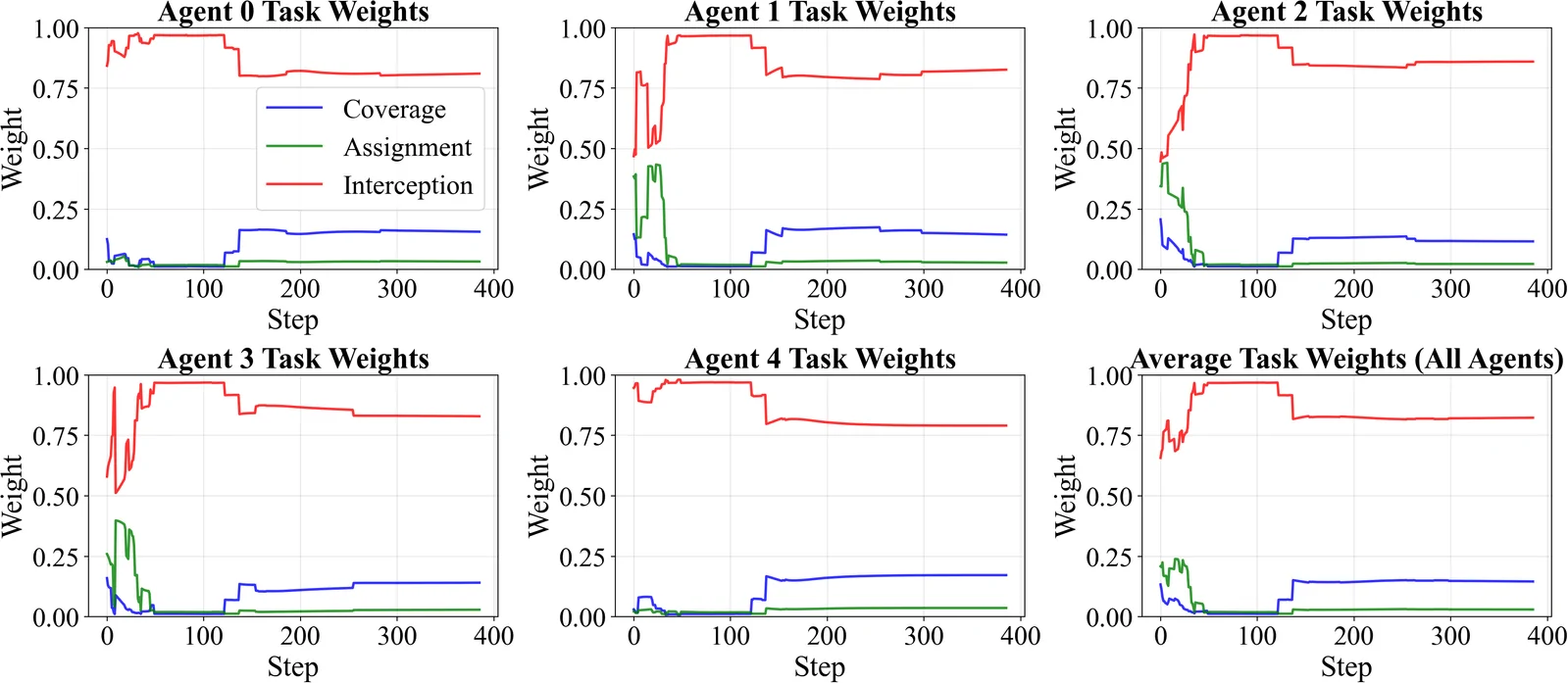
Real-time adaptive adjustment trajectory of reward weights.

To quantify the advantage, we compare against four fixed-weight schemes (detection:assignment:planning): 0.6:0.2:0.2, 0.2:0.6:0.2, 0.2:0.2:0.6, and 0.3:0.3:0.4. These four schemes are designed to represent qualitatively distinct strategies: single-subtask-dominant configurations (Schemes 1–3) and a balanced baseline (Scheme 4), thereby covering the key regions of the weight simplex without exhaustive enumeration. Results are shown in Table 7.

**Table 7:** Performance comparison between the fixed-weight and dynamic-weight schemes.

Ablated Module	Success	Avg. Intercept.	Avg. Time	Avg. Radius
W-MAPPO	94%	95.20%	72.34±5.18	8.12±1.08
Scheme 1	92%	93.60%	76.12±6.85	7.41±1.31
Scheme 2	89%	89.80%	75.34±7.12	7.53±1.06
Scheme 3	93%	95.00%	75.11±6.23	7.77±1.48
Scheme 4	89%	91.60%	79.54±8.35	7.35±1.44

W-MAPPO with dynamic weights outperforms all fixed-weight schemes across all metrics. Among fixed schemes, the path-planning-dominant configuration (Scheme 3) performs best, underscoring the importance of interception weight, yet the dynamic mechanism further improves performance by adapting to evolving task situations. The consistent advantage of W-MAPPO over all four configurations, including the best-performing Scheme 3, indicates that the benefit of dynamic weighting is robust across representative regions of the weight simplex and does not arise from any single fixed configuration being poorly specified.

(2) Necessity validation of LSTM-based temporal modeling

We evaluated performance under different LSTM time step settings (H∈{10,20,30,40}) and a baseline without LSTM (replaced by directly concatenating the last 5 observations as input, without recurrent encoding). Results are shown in Table 8.

**Table 8:** Evaluation effect comparison under different LSTM module settings.

LSTM Step Length	Success	Avg. Intercept.	Avg. Time	Avg. Radius
40	92%	92.60%	88.21±7.94	7.85±1.67
30	89%	89.80%	90.76±8.63	7.90±2.28
20	94%	95.20%	72.34±5.18	8.12±1.08
10	88%	91.20%	92.58±8.87	7.62±1.61
No LSTM	82%	84.20%	97.26±9.82	6.79±1.54

Removing the LSTM module and replacing it with a fixed-length sliding window (directly concatenating the last 5 observations) causes the largest performance drop (success rate: 82%, defense radius: 6.79±1.54), underscoring the importance of recurrent temporal modeling over simple concatenation. Among LSTM variants, performance peaks at *H* = 20 and declines for longer sequences (*H* = 30, 40), suggesting that excessive history length leads to overfitting at the expense of current-situation adaptability.

(3) Voronoi responsibility area collaborative detection mechanism

Table 9 compares W-MAPPO with a version that removes the Voronoi collaborative detection reward.

**Table 9:** Evaluation effect comparison of Voronoi responsibility area settings.

Detection mechanism	Success	Avg. Intercept.	Avg. Time	Avg. Radius
W-MAPPO	94%	95.20%	72.34±5.18	8.12±1.08
Remove Voronoi	85%	88.4%	92.21±8.11	7.07±1.58

Removing the Voronoi mechanism causes a 12% drop in success rate and 27% increase in interception time, underscoring its role in reducing detection redundancy and optimizing spatial coverage.

(4) Hyperparameter Sensitivity Analysis

The KL-regularization coefficient $\lambda_{\text{hist}}$ in Eq. (57) controls the smoothness of dynamic weight transitions (corresponding to condition (C3) in Theorem 1). We vary $\lambda_{\text{hist}}\in \{0,0.01,0.05,0.1,0.2,0.5\}$ in the 5 vs 5 scenario (100 test runs per setting). Results are shown in Table 10.

**Table 10:** Sensitivity analysis of the KL-regularization coefficient $\lambda_{hist}$ (5 vs 5 scenario).

$\lambda_{hist}$	Success	Avg. Intercept.	Avg. Time	Avg. Radius
0	90%	92.40%	78.45 ± 6.12	7.52 ± 1.15
0.01	92%	94.20%	75.92 ± 5.47	**8.18 ± 1.02**
0.05	**94%**	**95.20%**	**72.34 ± 5.18**	8.12 ± 1.08
0.1	93%	93.80%	74.51 ± 5.63	7.89 ± 1.12
0.2	92%	92.60%	73.86 ± 5.84	7.64 ± 1.18
0.5	87%	88.40%	82.67 ± 7.26	7.21 ± 1.34

The results exhibit an inverted-U relationship: when $\lambda_{\text{hist}}=0$, unconstrained weight updates cause oscillations that increase variance (std = 6.12 steps) and degrade success rate to 91%. The success rate and interception rate peak at $\lambda_{\text{hist}}=0.05$, while the average defense radius reaches its maximum at $\lambda_{\text{hist}}=0.01$ (8.18), indicating that lighter regularization preserves more aggressive outward interception behavior. The average interception time remains competitive across $\lambda_{\text{hist}}\in [0.05,0.2]$, suggesting moderate robustness in this range. Beyond 0.2, excessive regularization over-constrains weight adaptation; at $\lambda_{\text{hist}}=0.5$, weights become nearly static and success rate drops to 87%. This validates condition (C3) in Theorem 1 and indicates that $\lambda_{\text{hist}}\in [0.01,0.1]$ yields robust performance. Fig 8 visualizes this trend across all four metrics.

**Fig 8:**
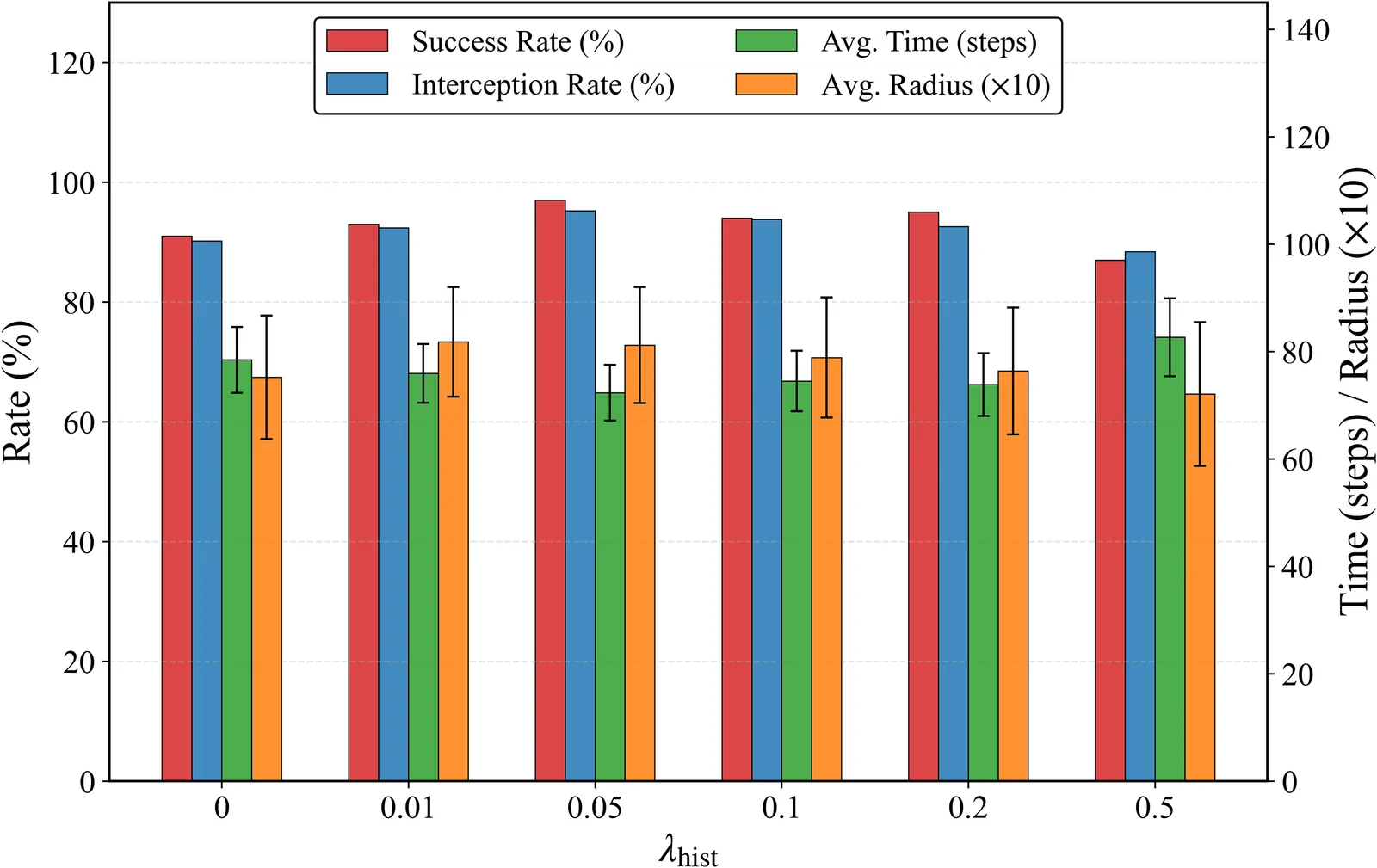
Sensitivity analysis of $\lambda_{hist}$. All four metrics exhibit an inverted-U pattern, peaking at $\lambda_{\text{hist}}=0.05$. The dashed vertical line marks the optimal setting.

(5) Robustness to Sensor and Communication Uncertainties

Real-world deployment faces sensor noise and communication failures not modeled in the baseline simulation. To assess the algorithm’s robustness to such uncertainties, we conducted a pilot experiment with domain randomization during training: randomly dropping 15% of detections (simulating sensor uncertainty) and 20% of neighbor messages (simulating communication packet loss) at each time step. The trained policy was then tested in the standard 5 vs 5 scenario (without noise injection during testing).

Results show that the robustness-trained W-MAPPO achieves 91% success rate (vs. 94% for the baseline trained under ideal conditions), demonstrating graceful degradation under sensor and communication uncertainties. This 3% performance drop is significantly smaller than the 12% drop observed when the baseline policy (trained without noise) is tested under the same noisy conditions (82% success rate). These preliminary results suggest that training-time noise injection can effectively prepare the policy for real-world uncertainties; further deployment considerations are discussed in the Engineering Deployment Considerations subsection.

### Case study

For the trained algorithm, we conducted multiple tests in the original interception task environment. Figs 9–11 illustrate representative simulation processes under different scales of threat targets, including both successful interceptions and a failure case that exposes capacity limitations. In these figures, the black rectangle denotes the interception task area, the red circle represents the central protected area, the blue dots represent agents, blue arrows indicate their headings, and green circles show their detection ranges. Gray X’s denote threat targets, which turn yellow upon detection and red upon interception, ceasing movement thereafter.

**Fig 9:**
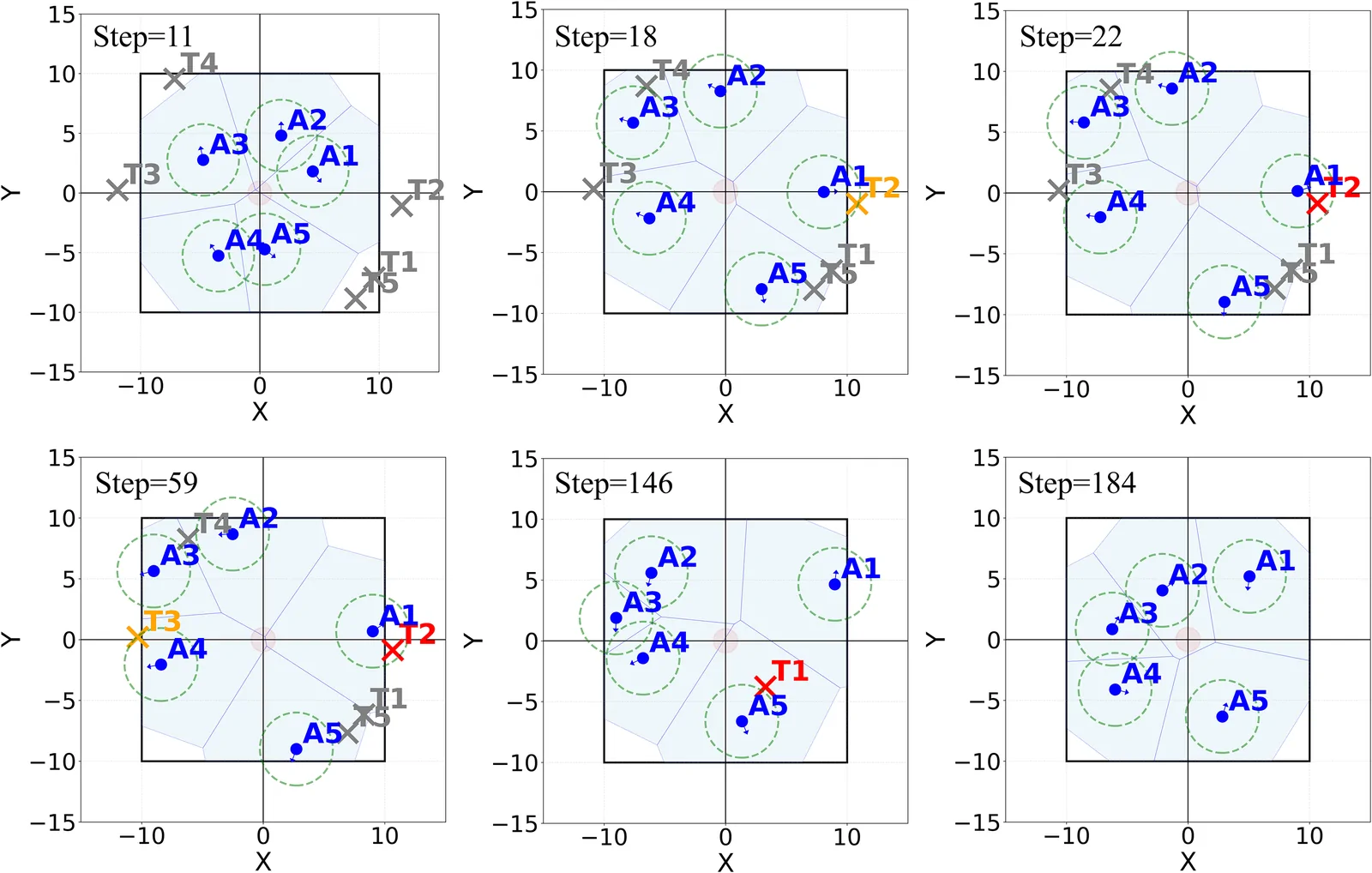
Typical interception process with 5 threat targets.

**Fig 10:**
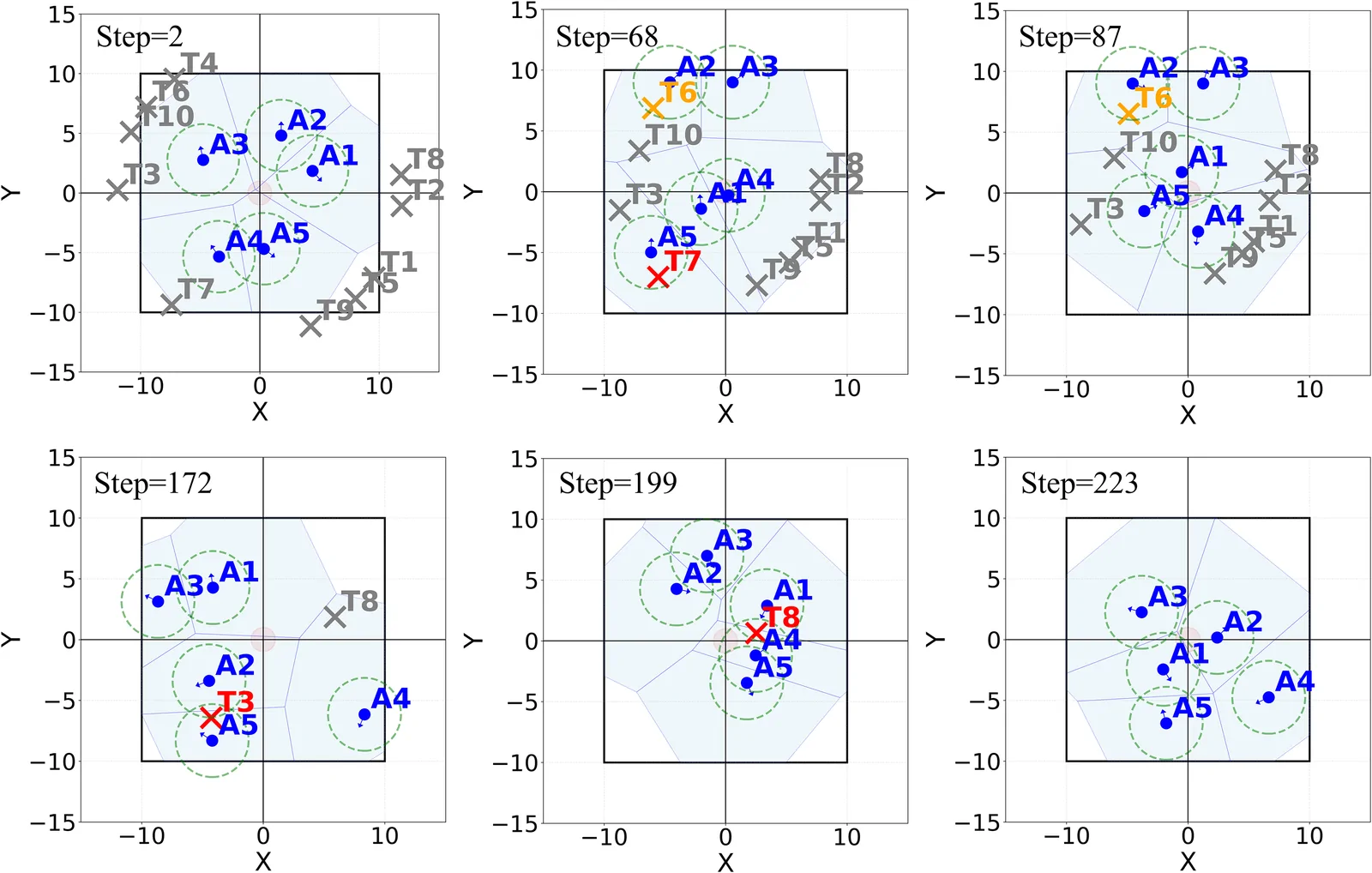
Typical interception process with 10 threat targets.

**Fig 11:**
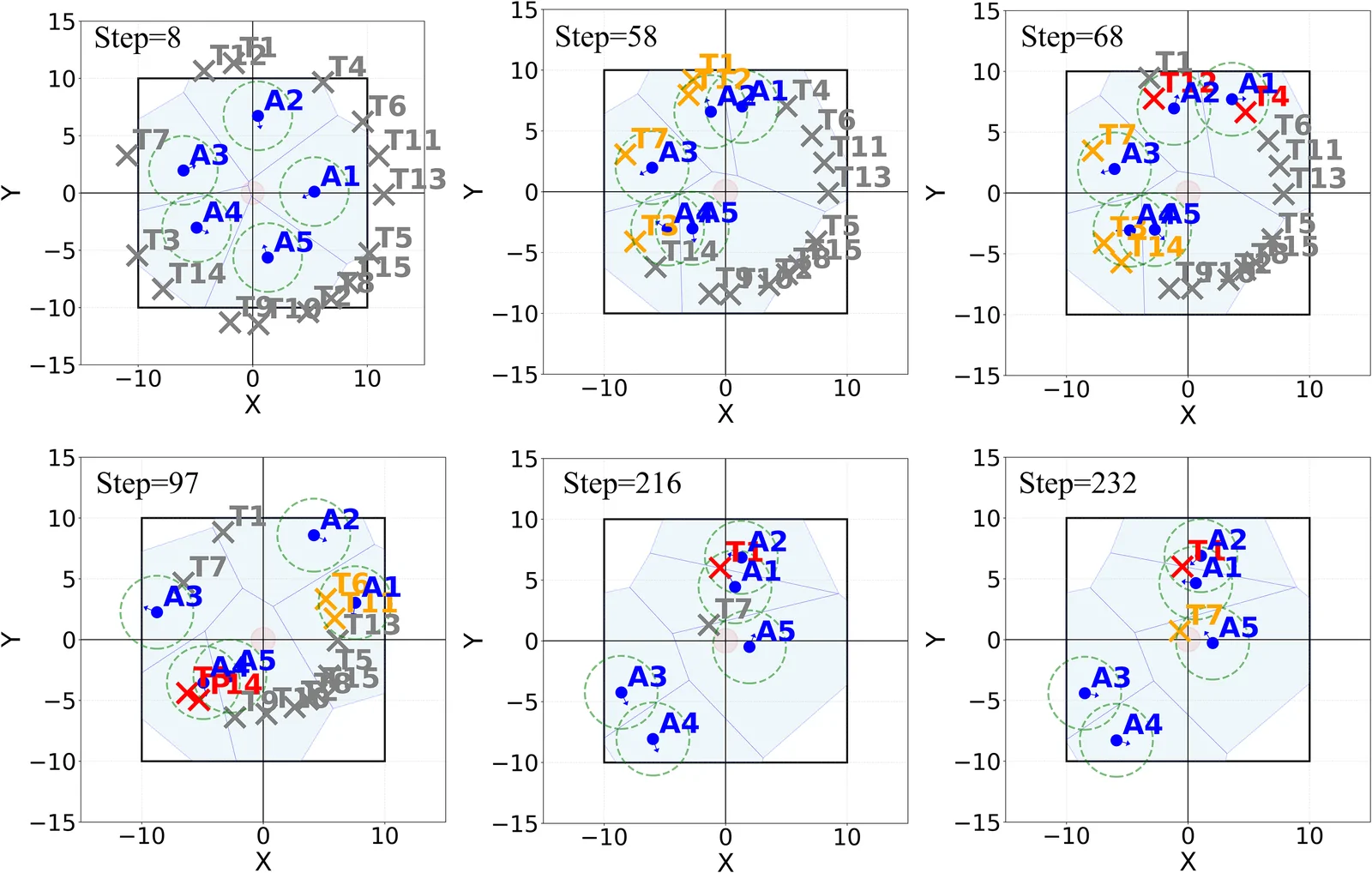
A representative failure case in the 5 vs 15 scenario where threats breach the protected area.

Fig 9 shows the scenario with five threat targets. In the initial phase, the MAS did not detect any threat targets, so task weights favored area exploration. To improve detection coverage, agents performed area exploration maneuvers based on Voronoi partition coordination (step = 11). When some agents (A1) detected threat targets, the task focus shifted to task assignment. Guided by distance rewards in task assignment, the agents maneuvered toward assigned targets (T2) (step = 18). As the agents continuously approached the task targets, the weight of path planning tasks increased, executing interception maneuvers against threat target movements until interception was completed (step = 22). Similarly, other agents collaboratively completed interception tasks (step = 59) until the last target was intercepted (step = 146). The MAS continued exploration without finding new targets, and the formation gradually tended toward uniform distribution (step = 184).

In higher-threat-density scenarios, we focus on how the algorithm handles difficult-to-intercept targets that exhibit sustained evasive behavior.

Fig 10 highlights the interception of threat target T3 in the 5 vs 10 scenario. T3 was initially located near (−12,0) (step = 2). As the MAS prioritized closer threats, T3 intruded into the task area unimpeded and randomly maneuvered below the *Y*-axis (step = 68). As agents approached, T3 stochastically entered feedback evasion mode and continued maneuvering downward along the *Y*-axis (step = 87). The dynamic task allocation mechanism reassigned agents to T3 once higher-priority targets were eliminated, and the pursuing agent successfully completed the interception (step = 172). After all remaining threats were neutralized (step = 199), the formation converged toward uniform spatial distribution (step = 223).

Fig 11 presents a representative failure case in the 5 vs 15 scenario, where the high threat density ultimately overwhelms the interception capacity. We focus on threat target T1, initially positioned near (−2,12) (step = 8). As multiple threats advanced toward the protected area, agent A2 simultaneously detected both T12 and T1 (step = 58). Under the distance-based task allocation mechanism, A2 prioritized T12 due to its closer proximity and executed the interception maneuver accordingly. This allocation decision triggered T1’s feedback evasion model, causing it to perform an outward escape trajectory (step = 68). T1 subsequently maneuvered beyond A2’s detection range (step = 97), temporarily evading surveillance. As the remaining agents continued distributed exploration, T1 was eventually rediscovered by A1 and successfully intercepted (step = 216). However, due to the excessive number and spatial dispersion of threat targets, the five agents were unable to maintain sufficient coverage, and several threats penetrated the central protected area, resulting in mission failure (step = 232). This case reveals the fundamental capacity limitation of the system: when the threat-to-agent ratio exceeds a critical threshold, the sequential nature of interception tasks creates temporal gaps that dispersed threats can exploit, even when the dynamic allocation mechanism operates correctly for individual engagements.

### Engineering deployment considerations

While the simulation results demonstrate W-MAPPO’s effectiveness under idealized conditions, real-world deployment requires addressing practical constraints not modeled in the simulation. This section outlines key engineering challenges that must be addressed in future work.

#### Simulation-to-reality gap.

Real sensors exhibit imperfect detection with false alarms, maritime communication suffers packet loss, and GPS accuracy degrades under jamming. To improve robustness, domain randomization during training is recommended: randomly dropping detections, injecting false positives, and adding noise to state estimates. Additionally, a hierarchical control architecture is suggested where W-MAPPO outputs desired velocity while a low-level controller handles platform-specific dynamics. As an initial step in this direction, the domain-randomization experiment reported in the robustness analysis above showed that training-time injection of sensor and communication noise narrows the performance loss under noisy testing from 12 to 3 percentage points, providing preliminary evidence that the learned coordination is not an artifact of idealized simulation.

#### Mapping to physical units and practical parameter values.

To anchor the simulation in physical terms, a reference scaling of one length unit to 100 m maps the task area to a 2×2 km^2^ sea region, the core zone to a 200 m-diameter protected area, and the agent speed bound $v_{c}^{\max}=1.0$ to 10 m/s, consistent with typical patrol USV speeds. The detection and communication radii then correspond to 300 m and 600 m, respectively, within the range of standard marine radar and radio links, while the intruder baseline and burst speeds map to 6 m/s and 8 m/s. Under a 100 ms control period typical of maritime autopilots, the measured 2.8 ms inference time on an RTX 4090 leaves ample margin; on embedded platforms of the Jetson class, INT8 quantization and pruning are expected to reduce per-step inference to well below 10 ms, and the modest inference gap relative to vanilla MAPPO (1.9 ms) indicates that the additional modules do not jeopardize real-time feasibility. These figures provide concrete reference values for migrating the framework from simulation to physical platforms.

#### Computational feasibility.

Table 6 reports 2.8 ms inference time on RTX 4090 GPU. For embedded platforms typical in USVs/UAVs, model compression techniques such as quantization and pruning may be necessary to meet real-time constraints. Given typical control periods of 100 ms in maritime applications, the computational requirements appear feasible, though hardware validation is needed.

#### Phased deployment roadmap.

A phased validation approach is recommended: starting with small-scale controlled experiments (2–3 agents vs. 1–2 targets), progressing to nearshore environments with moderate complexity, and finally scaling to open-water scenarios matching the simulation scale. Each phase should validate both interception performance and robustness to environmental uncertainties.

#### Limitations and future work.

Key limitations include: (1) **Simulation-only validation**: hardware experiments are essential to confirm sim-to-real transfer; (2) **Homogeneous agents**: extending to heterogeneous fleets with different sensor ranges and speeds requires further investigation, although the extension path is architectural rather than algorithmic—appending a per-agent capability vector to the observation preserves parameter sharing and permutation invariance, since heterogeneity then enters only through the observation; (3) **Static environment**: obstacle avoidance and dynamic hazards need to be incorporated; (4) **Communication constraints**: the current design assumes reliable message passing, while real networks may experience delays and bandwidth limitations; (5) **Lightweight coordination extensions**: explicit exchange of weight vectors among neighboring agents and maneuver-intensity-conditioned adaptive history horizons are natural extensions of the current modules, deferred to future work. Despite these limitations, the proposed framework provides a foundation for future deployment efforts.

## Conclusion

This paper addresses the joint optimization of distributed detection, dynamic task allocation, and interception path planning for multi-agent cooperative interception under partial observability, three tightly coupled subtasks that prior works either handle separately or solve under the assumption of global information. To this end, we propose W-MAPPO, a weighted coordinated multi-agent proximal policy optimization algorithm that unifies these subtasks within an end-to-end framework. The algorithm integrates a dynamic weight mechanism for multi-subtask coordination, an LSTM-based history-aware processor for temporal modeling, and a Voronoi-based collaborative detection module for spatial coverage optimization. A stability characterization establishes an approximate stationary-region bound for the dynamic weight mechanism, whose residual size is governed by the KL regularization coefficient.

Simulation results demonstrate that W-MAPPO achieves a 94% success rate in the 5 vs 5 scenario and maintains robust performance as threat scale increases (78% in 5 vs 10, 67% in 5 vs 15), consistently outperforming seven representative MARL baselines. Ablation studies confirm that each module contributes meaningfully, with the success rate degrading by 5–12 percentage points upon removal or replacement of any single component.

Current limitations include the assumptions of homogeneous agents and reliable communication. Future work will extend to heterogeneous multi-platform systems with role specialization, investigate robustness under communication constraints and adversarial countermeasures, and validate the framework in hardware-in-the-loop testbeds.

## Supporting information

S1 Code Source code of the W-MAPPO framework and of all baseline algorithms, including the simulation environment, training scripts, and evaluation scripts. (ZIP)

S1 Dataset Raw experimental data underlying Fig 5 and Tables 5, 7–10, organized per experiment and per algorithm: multi-algorithm comparison logs (Table 5), fixed-weight scheme logs (Table 7), LSTM setting logs (Table 8), Voronoi ablation logs (Table 9), KL-coefficient sensitivity logs (Table 10), and training curves (Fig 5). (ZIP)
