# Supplementary material for: Joint optimization of detection, task allocation, and path planning for multi-agent cooperative interception under partial observability
Source: PLoS One. 2026 Oct 13;21(10):e0359071. doi: 10.1371/journal.pone.0359071 (PMC13660932; doi:10.1371/journal.pone.0359071)
Supplement: Peer review history

# Peer review: Decision Letter 0

**DOI:** 10.1371/journal.pone.0359071.r001

**Authors:** Qingan Qiu

5 Aug 2026

Dear Dr. Liu,

Thank you for submitting your manuscript to PLOS ONE. After careful consideration, we feel that it has merit but does not fully meet PLOS ONE’s publication criteria as it currently stands. Therefore, we invite you to submit a revised version of the manuscript that addresses the points raised during the review process.

Please submit your revised manuscript by Oct 04 2026 11:59PM. If you will need more time than this to complete your revisions, please reply to this message or contact the journal office at plosone@plos.org. When you're ready to submit your revision, log on to https://www.editorialmanager.com/pone/ and select the 'Submissions Needing Revision' folder to locate your manuscript file.

- A letter that responds to each point raised by the academic editor and reviewer(s). You should upload this letter as a separate file labeled 'Response to Reviewers'.
- A marked-up copy of your manuscript that highlights changes made to the original version. You should upload this as a separate file labeled 'Revised Manuscript with Track Changes'.
- An unmarked version of your revised paper without tracked changes. You should upload this as a separate file labeled 'Manuscript'.

If you would like to make changes to your financial disclosure, please include your updated statement in your cover letter. Guidelines for resubmitting your figure files are available below the reviewer comments at the end of this letter.

If applicable, we recommend that you deposit your laboratory protocols in protocols.io to enhance the reproducibility of your results. Protocols.io assigns your protocol its own identifier (DOI) so that it can be cited independently in the future. For instructions see: https://journals.plos.org/plosone/s/submission-guidelines#loc-laboratory-protocols. Additionally, PLOS ONE offers an option for publishing peer-reviewed Lab Protocol articles, which describe protocols hosted on protocols.io. Read more information on sharing protocols at https://plos.org/protocols?utm_medium=editorial-email&utm_source=authorletters&utm_campaign=protocols.

As the corresponding author, your ORCID iD is verified in the submission system and will appear in the published article. PLOS supports the use of ORCID, and we encourage all coauthors to register for an ORCID iD and use it as well. Please encourage your coauthors to verify their ORCID iD within the submission system before final acceptance, as unverified ORCID iDs will not appear in the published article. *Only*  the individual author can complete the verification step; PLOS staff *cannot*  verify ORCID iDs on behalf of authors.

We look forward to receiving your revised manuscript.

Kind regards,

Qingan Qiu

Academic Editor

PLOS One

Journal Requirements:

When submitting your revision, we need you to address these additional requirements.

1. Please ensure that your manuscript meets PLOS ONE's style requirements, including those for file naming. The PLOS ONE style templates can be found at

https://journals.plos.org/plosone/s/file?id=wjVg/PLOSOne_formatting_sample_main_body.pdf and

https://journals.plos.org/plosone/s/file?id=ba62/PLOSOne_formatting_sample_title_authors_affiliations.pdf

2. Please note that PLOS One has specific guidelines on code sharing for submissions in which author-generated code underpins the findings in the manuscript. In these cases, we expect all author-generated code to be made available without restrictions upon publication of the work. Please review our guidelines at https://journals.plos.org/plosone/s/materials-and-software-sharing#loc-sharing-code and ensure that your code is shared in a way that follows best practice and facilitates reproducibility and reuse.

3. Thank you for stating the following in the Acknowledgments Section of your manuscript:

“This work was supported by the National Natural Science Foundation of China 62506378.”

We note that you have provided funding information that is not currently declared in your Funding Statement. However, funding information should not appear in the Acknowledgments section or other areas of your manuscript. We will only publish funding information present in the Funding Statement section of the online submission form.

Please remove any funding-related text from the manuscript and let us know how you would like to update your Funding Statement. Currently, your Funding Statement reads as follows:

“the National Natural Science Foundation of China 62506378”

Please include your amended statements within your cover letter; we will change the online submission form on your behalf.

4. Thank you for stating the following financial disclosure:

“the National Natural Science Foundation of China 62506378”

Please state what role the funders took in the study.  If the funders had no role, please state: "The funders had no role in study design, data collection and analysis, decision to publish, or preparation of the manuscript."

If this statement is not correct you must amend it as needed.

Please include this amended Role of Funder statement in your cover letter; we will change the online submission form on your behalf.

5. PLOS requires an ORCID iD for the corresponding author in Editorial Manager on papers submitted after December 6th, 2016. Please ensure that you have an ORCID iD and that it is validated in Editorial Manager. To do this, go to ‘Update my Information’ (in the upper left-hand corner of the main menu), and click on the Fetch/Validate link next to the ORCID field. This will take you to the ORCID site and allow you to create a new iD or authenticate a pre-existing iD in Editorial Manager.

6. Please include captions for your Supporting Information files at the end of your manuscript, and update any in-text citations to match accordingly. Please see our Supporting Information guidelines for more information: http://journals.plos.org/plosone/s/supporting-information.

7. We are unable to open your Supporting Information file “refs.bib”. Please kindly revise as necessary and re-upload.

8. If the reviewer comments include a recommendation to cite specific previously published works, please review and evaluate these publications to determine whether they are relevant and should be cited. There is no requirement to cite these works unless the editor has indicated otherwise.

[Note: HTML markup is below. Please do not edit.]

Reviewers' comments:

Reviewer's Responses to Questions

**Comments to the Author**

1. Is the manuscript technically sound, and do the data support the conclusions?

Reviewer #1: Yes

Reviewer #2: Yes

**********

2. Has the statistical analysis been performed appropriately and rigorously?

Reviewer #1: Yes

Reviewer #2: Yes

**********

3. Have the authors made all data underlying the findings in their manuscript fully available?

The PLOS Data policy

Reviewer #1: Yes

Reviewer #2: Yes

**********

4. Is the manuscript presented in an intelligible fashion and written in standard English?

Reviewer #1: Yes

Reviewer #2: Yes

**********

Reviewer #1: This work addresses coupled multi-agent interception under partial observability, formulating detection, task allocation, path planning as a joint Dec-POMDP optimization problem. It proposes W-MAPPO with three innovations: a policy ratio predictor for dynamic subtask weight balancing, LSTM-attention history processor modeling target evasive trajectories, and Voronoi collaborative detection for spatial coverage coordination. In all, this work is enriching. However, it needs to be further improved. Specific comments are as below:

1) The current policy ratio predictor only relies on single-step local observation to generate weights, lacking inter-agent weight coordination.

2) Fixed history length cannot adapt to target maneuver complexity; static sequence length wastes computation for slow-evasion targets and loses critical trajectory information for high-speed evaders.

3) All experiments adopt identical agent kinematic parameters, limiting practical deployment.

Reviewer #2: This manuscript addresses an important problem with a well-designed framework and promising experimental results. However, the novelty of individual components relative to existing work, the practical tractability of the Dec-POMDP formulation, and the real-world deployment feasibility need to be more clearly established. I recommend major revision to address these concerns before considering for publication.

1.The manuscript introduces a complex framework (W-MAPPO) with multiple components, but the novelty of each individual component relative to existing literature is not clearly articulated. For instance, the use of LSTM for temporal modeling in multi-agent settings has been explored in prior work (e.g., LSTM-based approaches in Dec-POMDPs), and Voronoi-based coverage coordination is a well-established technique in multi-agent systems. The authors should explicitly compare their Policy Ratio Predictor with existing policy gradient methods and clarify what specific design choices make W-MAPPO novel beyond the straightforward integration of these components. Additionally, the "weighted" aspect of W-MAPPO should be explained—what does the weighting scheme represent, and how does it differ from standard multi-task weighting approaches?

2.The formulation as a "coupled Dec-POMDP" is mentioned but not elaborated in the abstract. Dec-POMDPs are notoriously computationally intractable (NEXP-complete), and the authors should clarify what assumptions or simplifications are adopted to make the problem tractable.

3.Author claim "practical potential" but does not discuss the transition from simulation to real-world deployment. Authors could give more practical values.

4.More related references should be added in this paper, for example, Condition-based maintenance strategy for redundant systems with arbitrary structures using improved reinforcement learning. Reliability Engineering & System Safety, 2022, 225: 108643. Joint optimization of quality control and maintenance policy for a production system with quality-dependent failures, Expert Systems With Applications, 2025, 272: 126800. Joint optimization of maintenance policy and two-way stock transshipments policy for balanced systems, Reliability Engineering and System Safety, 2025, 264: 111345. Multi-level adaptive risk control: synergistic maintenance-termination optimization with online parameter learning. Reliability Engineering & System Safety, 2025: 112138.

**********

what does this mean?). If published, this will include your full peer review and any attached files.

If you choose “no”, your identity will remain anonymous but your review may still be made public.

**Do you want your identity to be public for this peer review?**  For information about this choice, including consent withdrawal, please see our Privacy Policy

Reviewer #1: No

Reviewer #2: No

**********

[NOTE: If reviewer comments were submitted as an attachment file, they will be attached to this email and accessible via the submission site. Please log into your account, locate the manuscript record, and check for the action link "View Attachments". If this link does not appear, there are no attachment files.]

To ensure your figures meet our technical requirements, please review our figure guidelines: https://journals.plos.org/plosone/s/figures

You may also use PLOS’s free figure tool, NAAS, to help you prepare publication quality figures: https://journals.plos.org/plosone/s/figures#loc-tools-for-figure-preparation.

NAAS will assess whether your figures meet our technical requirements by comparing each figure against our figure specifications.

# Author comment: Author response to Decision Letter 1

**DOI:** 10.1371/journal.pone.0359071.r002

1 Sep 2026

Response to Reviewers

Manuscript ID: PONE-D-26-36856

Title: Joint Optimization of Detection, Task Allocation, and Path Planning for Multi-Agent Cooperative Interception under Partial Observability

Journal: PLOS ONE

Dear Dr. Qiu and Reviewers,

We sincerely thank the Academic Editor and both reviewers for the careful reading of our manuscript and for the constructive comments. We have revised the manuscript accordingly. All changes are marked in the "Revised Manuscript with Track Changes" file (produced with latexdiff). Line numbers cited below refer to the margin line numbers printed in the unmarked revised manuscript (the "Manuscript" PDF), which are numbered continuously from the first page of the Introduction (the title and abstract pages are unnumbered). We have also addressed all journal requirements, as detailed below.

Part 1: Responses to Journal Requirements

Requirement 1 (Style requirements / file naming).

We have checked the manuscript against the PLOS ONE style templates and adjusted the formatting and file naming accordingly. The manuscript continues to use the official PLOS LaTeX template.

Requirement 2 (Code sharing).

We fully agree with PLOS's code-sharing policy. The simulation code and the experimental data underlying the findings have been deposited in a public repository: https://github.com/FengYeXuanLv/_code_src. The Data Availability Statement in the revised manuscript has been updated accordingly (see "Data availability" section, line 814).

Requirement 3 (Funding information).

We thank the editor for pointing this out. We have removed the funding-related text from the manuscript (the "Funding" section and the funding sentence in the Acknowledgments have been deleted). The amended Funding Statement is provided in the cover letter:

"This work was supported by the National Natural Science Foundation of China (Grant No. 62506378)."

Requirement 4 (Role of funder).

The following statement, exactly as requested by the journal, is included in the cover letter:

"The funders had no role in study design, data collection and analysis, decision to publish, or preparation of the manuscript."

Requirement 5 (ORCID iD).

The corresponding author's ORCID iD (0009-0000-2647-8000) has been validated in Editorial Manager.

Requirement 6 (Supporting information captions).

We have added a "Supporting information" section at the end of the revised manuscript (line 821) containing captions for all Supporting Information files (S1 Code and S1 Dataset), and in-text citations to the Supporting information have been added where appropriate.

Requirement 7 (refs.bib file).

We apologize for the confusion. refs.bib is a component of the LaTeX source (the reference list compiles into the manuscript PDF) and was uploaded as a Supporting Information file by mistake. We have removed it from the Supporting Information list. If the journal still requires the reference list as a separate file, we are happy to provide it as a plain-text file.

Requirement 8 (Suggested citations).

We have evaluated all works suggested by the reviewers and found the four works recommended by Reviewer #2 relevant to the joint-optimization theme of our study. All four have been cited and discussed in the Introduction (see also our response to Reviewer #2, Comment 4).

Part 2: Responses to Reviewer #1

We thank Reviewer #1 for the positive assessment ("this work is enriching") and for the three constructive comments, which we address as follows.

Comment 1

*"The current policy ratio predictor only relies on single-step local observation to generate weights, lacking inter-agent weight coordination."*

Response: We agree that the original manuscript did not explicitly describe how inter-agent coordination constrains the generated weights, and we thank the reviewer for this observation. We would like to clarify that two coordination channels exist in the current design, and we have now made them explicit in the revised manuscript:

1. Observation-level context: Although the predictor consumes the agent's own observation, this observation embeds the states and detection information of up to K_max neighbors within the communication range, together with the Voronoi zone encoding. The generated weights are therefore conditioned on the local multi-agent context (Policy Ratio Predictor subsection, new clarifying paragraph, line 346).

2. Reward-level coordination: Allocation conflicts are explicitly penalized by the distributed competition penalty (Eq. 27), which discourages neighboring agents from concentrating on the same target.

We also fully agree that explicit exchange of raw weight vectors among neighbors is a natural lightweight extension (it would add only three observation dimensions), and we have identified it as such in the Limitations (item (5), line 788).

Comment 2

*"Fixed history length cannot adapt to target maneuver complexity; static sequence length wastes computation for slow-evasion targets and loses critical trajectory information for high-speed evaders."*

Response: We thank the reviewer for this insightful comment. Two points deserve clarification, both of which are now stated explicitly in the revised manuscript (History-Aware Processor subsection, new paragraph, line 388):

1. The attention mechanism provides soft temporal adaptation within the fixed window. Frames relevant to the current maneuver regime receive higher attention weights, while redundant or stale frames are down-weighted, so the effective receptive field adapts to target behavior even though the window length H is fixed.

2. The existing sensitivity study demonstrates robustness to window length. The LSTM step-length comparison (Table 7) shows that performance remains within a narrow band (88%–94% success rate) for H ∈ {10, 20, 30, 40}, and that all recurrent settings substantially outperform the no-LSTM baseline, indicating that the design is not fragile to the fixed-horizon choice across the maneuver regimes covered by the hybrid threat model.

We agree that fully adaptive horizon selection conditioned on an estimated target maneuver intensity would be a valuable extension, and we have added it to the Limitations (item (5), line 788) as future work.

Comment 3

*"All experiments adopt identical agent kinematic parameters, limiting practical deployment."*

Response: We agree. The homogeneous-agent setting is a deliberate design choice that enables parameter sharing and permutation invariance; we have now clarified in the Limitations (item (2), line 784) that extending to heterogeneous fleets is an architectural rather than algorithmic change—a per-agent capability vector appended to the observation preserves parameter sharing, since heterogeneity then enters only through the observation. In addition, to strengthen the practical-deployment discussion, we have added a "Mapping to Physical Units and Practical Parameter Values" subsection in the Engineering Deployment Considerations (line 759), which maps the dimensionless simulation parameters to physical quantities (task area 2×2 km², agent speed 10 m/s, detection/communication radii 300/600 m, etc.) and discusses embedded-platform feasibility (Jetson-class, <10 ms inference under a 100 ms control period). We also note that the domain-randomization experiment already reported in the manuscript shows graceful degradation under sensor and communication uncertainties (91% vs. 94%), which we now explicitly connect to the sim-to-real discussion. We respectfully suggest that a dedicated heterogeneous-agent experiment, while valuable, is beyond the scope of the present revision and is planned for our future work on heterogeneous multi-platform systems.

Part 3: Responses to Reviewer #2

We thank Reviewer #2 for the careful evaluation and for recommending major revision. We address the four concerns below.

Comment 1

*"The novelty of each individual component relative to existing literature is not clearly articulated... The authors should explicitly compare their Policy Ratio Predictor with existing policy gradient methods... Additionally, the 'weighted' aspect of W-MAPPO should be explained—what does the weighting scheme represent, and how does it differ from standard multi-task weighting approaches?"*

Response: We thank the reviewer for this important comment, which has substantially improved the positioning of our work. We have added a dedicated paragraph in the Related Work section (line 105) that (i) reviews the training-level weighting families---gradient-based adaptation, homoscedastic uncertainty weighting, and gradient surgery---drawing on a recent comprehensive survey in IEEE TPAMI (Vandenhende et al., 2022) and recent conflict-avoidant multi-objective RL work in IEEE Transactions on Information Theory (Wang et al., 2025)---and (ii) clarifies the fundamental difference of our design:

1. The weights are part of the policy, not of the optimizer. GradNorm/uncertainty/PCGrad operate at the training level, treating the weight vector as an optimization coefficient. In W-MAPPO, the weights are generated at execution time from local observations: each agent continuously reallocates its own attention among detection, allocation, and interception as the situation evolves, and the weights are regularized toward historically consistent values by the KL-based history loss (Eq. 51). In other words, our mechanism converts static loss-level balancing into an execution-time, observation-conditioned, history-regularized coordination mechanism.

2. The "Weighted" aspect. We have clarified that "Weighted" refers to the dynamic convex combination of the three subtask rewards, R^i(t) = ρ_det·R_det + ρ_ass·R_ass + ρ_int·R_int, with weights on the probability simplex (Eq. 12), generated per agent and per time step.

3. Per-component novelty. We have clarified that the role of the LSTM module is not memory augmentation of the policy (as in recurrent and sequence-model variants, citing the recent survey by Wen et al. 2023 in Frontiers of Computer Science), but a consistency regularizer that infers temporally coherent weight and value estimates from the interaction history; and that the Voronoi module departs from classical centralized coverage controllers in that the cell area is approximated from local samples within the communication range under partial observability and is used as a reward signal rather than a control law. The novelty of W-MAPPO therefore lies in the integration of these roles rather than in the individual building blocks.

Comment 2

*"The formulation as a 'coupled Dec-POMDP' is mentioned but not elaborated in the abstract. Dec-POMDPs are notoriously computationally intractable (NEXP-complete), and the authors should clarify what assumptions or simplifications are adopted to make the problem tractable."*

Response: We thank the reviewer for this point. We have added a dedicated tractability paragraph in the Dec-POMDP formulation section (line 216) that explicitly acknowledges NEXP-completeness and lists the five mechanisms that make the problem tractable in practice:

1. factored joint policy under CTDE, so execution-time complexity grows linearly in M;

2. parameter sharing, compressing the joint policy space to a single policy;

3. a centralized critic used only during training;

4. a two-timescale decision structure reducing the effective branching factor;

5. policy-gradient optimization that provides approximate rather than exact optimality, consistent with the approximate stationary-region analysis in the W-MAPPO section.

The abstract has been amended accordingly to state that the coupled Dec-POMDP is "made computationally tractable through factored decentralized policies, parameter sharing, and centralized training with decentralized execution."

Comment 3

*"Author claim 'practical potential' but does not discuss the transition from simulation to real-world deployment. Authors could give more practical values."*

Response: We thank the reviewer for this suggestion. We respectfully note that the submitted manuscript contained an "Engineering Deployment Considerations" subsection (with simulation-to-reality gap, computational feasibility, and a phased deployment roadmap) and a domain-randomization robustness experiment; however, we agree these were insufficiently quantitative and prominent. In the revised manuscript we have:

1. added the "Mapping to Physical Units and Practical Parameter Values" subsection (line 759) providing concrete physical values (2×2 km² task area, 10 m/s agent speed, 300/600 m detection/communication radii, 6–8 m/s intruder speeds, 100 ms control period, <10 ms embedded inference);

2. explicitly connected the domain-randomization experiment (91% vs. 94% success, and the reduction of the noise-induced performance loss from 12 to 3 percentage points) to the sim-to-real discussion;

3. retained the phased deployment roadmap, which progresses from small-scale controlled experiments (2--3 agents vs. 1--2 targets) through nearshore environments to open-water scenarios matching the simulation scale.

Comment 4

*"More related references should be added in this paper, for example, [four references on joint optimization in reliability engineering]."*

Response: We thank the reviewer for these suggestions. We have evaluated all four works and found them relevant to the central theme of our study—jointly optimizing coupled decision variables rather than treating them in isolation. All four have now been cited and discussed in the Introduction (line 27):

*"...in reliability engineering, reinforcement-learning-based condition-based maintenance for redundant systems with arbitrary structures [Yang et al. 2022], joint optimization of quality control and maintenance policies under quality-dependent failures [Wang et al. 2025], joint maintenance and two-way stock transshipment policies for balanced systems [Wang et al. 2025], and multi-level adaptive risk control with online parameter learning [Ma et al. 2026] all demonstrate the benefits of integrated optimization of interdependent objectives, further motivating the coupled formulation adopted in this study."*

Part 4: Additional corrections made in the revised manuscript

Beyond the reviewer comments, we corrected the following issues found during revision:

1. Duplicate Acknowledgments section removed (the manuscript contained two Acknowledgments sections; they have been merged into one).

2. Funding text removed from the manuscript per journal policy (see Requirement 3 above).

3. Broken cross-references fixed (three `\ref` commands pointing to undefined labels have been replaced with explicit textual references).

4. Data Availability Statement updated to comply with the PLOS data and code policy (see Requirement 2 above).

5. Supporting information section added with captions for all SI files (see Requirement 6 above).

6. Conclusion wording aligned with the theoretical analysis: the claim of "approximate convergence guarantees" has been reworded to "a stability characterization [that] establishes an approximate stationary-region bound," consistent with the statement of the theorem, to avoid overstating the theoretical contribution (line 800).

7. Statistical phrasing corrected: "decreases by 27%" was changed to "decreases by 27 percentage points" (and likewise for other percentage differences), for precision.

8. The "eight learning-based algorithms" phrase in the training-convergence discussion has been clarified as "W-MAPPO plus the seven MARL baselines."

We believe the revised manuscript has been substantially improved by these changes and hope that it now meets the criteria for publication in PLOS ONE. We thank the editor and reviewers once again for their time and valuable feedback.

Sincerely,

Zhikun Liu (on behalf of all authors)

Naval University of Engineering, Wuhan, China

Attachment Submitted filename: Response_to_Reviewers.docx

# Peer review: Decision Letter 1

**DOI:** 10.1371/journal.pone.0359071.r003

**Authors:** Qingan Qiu

10 Sep 2026

Joint Optimization of Detection, Task Allocation, and Path Planning for Multi-Agent Cooperative Interception under Partial Observability

PONE-D-26-36856R1

Dear Dr. Liu,

We’re pleased to inform you that your manuscript has been judged scientifically suitable for publication and will be formally accepted for publication once it meets all outstanding technical requirements.

Within one week, you’ll receive an e-mail detailing the required amendments. When these have been addressed, you’ll receive a formal acceptance letter and your manuscript will be scheduled for publication.

An invoice will be generated when your article is formally accepted. Please note, if your institution has a publishing partnership with PLOS and your article meets the relevant criteria, all or part of your publication costs will be covered. Please make sure your user information is up-to-date by logging into Editorial Manager at Editorial Manager® and clicking the ‘Update My Information' link at the top of the page. For questions related to billing, please contact billing support.

If your institution or institutions have a press office, please notify them about your upcoming paper to help maximize its impact. If they’ll be preparing press materials, please inform our press team as soon as possible -- no later than 48 hours after receiving the formal acceptance. Your manuscript will remain under strict press embargo until 2 pm Eastern Time on the date of publication. For more information, please contact onepress@plos.org.

Kind regards,

Qingan Qiu

Academic Editor

PLOS One

Additional Editor Comments (optional):

Reviewers' comments:

Reviewer's Responses to Questions

**Comments to the Author**

Reviewer #1: All comments have been addressed

Reviewer #2: (No Response)

**********

2. Is the manuscript technically sound, and do the data support the conclusions?

Reviewer #1: Yes

Reviewer #2: (No Response)

**********

3. Has the statistical analysis been performed appropriately and rigorously?

Reviewer #1: Yes

Reviewer #2: (No Response)

**********

4. Have the authors made all data underlying the findings in their manuscript fully available?

The PLOS Data policy

Reviewer #1: Yes

Reviewer #2: (No Response)

**********

5. Is the manuscript presented in an intelligible fashion and written in standard English?

Reviewer #1: Yes

Reviewer #2: (No Response)

**********

Reviewer #1: this version is ok. the authors have prepared responses well and the revised version is satisfactory.

Reviewer #2: (No Response)

**********

what does this mean?). If published, this will include your full peer review and any attached files.

If you choose “no”, your identity will remain anonymous but your review may still be made public.

**Do you want your identity to be public for this peer review?**  For information about this choice, including consent withdrawal, please see our Privacy Policy

Reviewer #1: No

Reviewer #2: No

**********

# Editor report: Acceptance letter

**DOI:** 10.1371/journal.pone.0359071.r004

**Authors:** Qingan Qiu

PONE-D-26-36856R1

PLOS One

Dear Dr. Liu,

I'm pleased to inform you that your manuscript has been deemed suitable for publication in PLOS One. Congratulations! Your manuscript is now being handed over to our production team.

At this stage, our production department will prepare your paper for publication. This includes ensuring the following:

* All references, tables, and figures are properly cited

* All relevant supporting information is included in the manuscript submission,

* There are no issues that prevent the paper from being properly typeset

You will receive further instructions from the production team, including instructions on how to review your proof when it is ready. Please keep in mind that we are working through a large volume of accepted articles, so please give us a few days to review your paper and let you know the next and final steps.

Lastly, if your institution or institutions have a press office, please let them know about your upcoming paper now to help maximize its impact. If they'll be preparing press materials, please inform our press team within the next 48 hours. Your manuscript will remain under strict press embargo until 2 pm Eastern Time on the date of publication. For more information, please contact onepress@plos.org.

You will receive an invoice from PLOS for your publication fee after your manuscript has reached the completed accept phase. If you receive an email requesting payment before acceptance or for any other service, this may be a phishing scheme. Learn how to identify phishing emails and protect your accounts at https://explore.plos.org/phishing.

If we can help with anything else, please email us at customercare@plos.org.

Thank you for submitting your work to PLOS One and supporting open access.

Kind regards,

PLOS One Editorial Office Staff

on behalf of

Dr. Qingan Qiu

Academic Editor

PLOS One
